# In Silico Identification and Characterization of Spiro[1,2,4]triazolo[1,5-*c*]quinazolines as Diacylglycerol Kinase α Modulators

**DOI:** 10.3390/molecules30112324

**Published:** 2025-05-26

**Authors:** Lyudmyla Antypenko, Kostiantyn Shabelnyk, Oleksii Antypenko, Mieko Arisawa, Oleksandr Kamyshnyi, Valentyn Oksenych, Serhii Kovalenko

**Affiliations:** 1 Independent Researcher, 11 Lamana Str., 69063 Zaporizhzhia, Ukraine; 2 Department of Pharmaceutical, Organic and Bioorganic Chemistry, Zaporizhzhia State Medical and Pharmaceutical University, 26 Maria Prymachenko Blvd., 69035 Zaporizhzhia, Ukraine; kshabelnik@gmail.com (K.S.); antypenkoan@gmail.com (O.A.); 3 Graduate School of Bioresources and Bioenvironmental Sciences, Kyushu University, 744 W5-674, Motooka Nishi-ku, Fukuoka 819-0395, Japan; arisawa@agr.kyushu-u.ac.jp; 4 Department of Microbiology, Virology and Immunology, I. Horbachevsky Ternopil State Medical University, 46001 Ternopil, Ukraine; 5 Department of Clinical Science, University of Bergen, Langes Gate 1-3, 5020 Bergen, Norway; 6 Institute of Chemistry and Geology, Oles Honchar Dnipro National University, 72 Nauky Ave., 49010 Dnipro, Ukraine; kovalenkosergiy@gmail.com

**Keywords:** diacylglycerol kinase, molecular docking, spirotriazoloquinazolines, structure–activity relationships, computational drug design

## Abstract

A new class of spiro[1,2,4]triazolo[1,5-*c*]quinazoline derivatives is presented as promising modulators of diacylglycerol kinase α (DGK-α), a target implicated in cancer, neurological disorders, and immune dysfunction. Through structure-based computational design using the CB-Dock2 platform with human DGK-α (PDB ID: 6IIE), 40 novel compounds were systematically evaluated along with established inhibitors (ritanserin, R59022, R59949, BMS502, and (5*Z*,2*E*)-CU-3) across five distinct binding pockets. Several compounds demonstrated binding profiles at the level of or surpassing the reference compounds. The physicochemical analysis revealed balanced drug-like properties with favorable molecular weights (252–412 g/mol) and appropriate three-dimensionality. The toxicological assessment indicated reassuring safety profiles with predicted LD_50_ values of 1000–2000 mg/kg and minimal hepatotoxicity, carcinogenicity, and mutagenicity potential. Notably, compound **33** (adamantyl-substituted) emerged as exceptionally promising, exhibiting strong binding affinity, moderate solubility, and selective CYP inhibition patterns that minimize drug–drug interaction risks. Detailed molecular interaction mapping identified critical binding determinants, including strategic hydrogen bonding with TRP151, GLU166, and ARG126. The multidimensional evaluation identified compounds **13**, **18**, **33**, and **40** as particularly promising candidates that balance potent target engagement with favorable pharmaceutical profiles, establishing this scaffold as a valuable platform for developing next-generation therapeutics targeting DGK-α -mediated signaling pathways.

## 1. Introduction

Diacylglycerol kinase α (DGK-α) has emerged as a promising therapeutic target, playing crucial roles in cell proliferation, T-cell responses, and viral infections. Recent studies have demonstrated that DGK-α inhibition could enhance antitumor immunity and potentially serve as a strategy for treating viral infections such as HIV and HBV [1].

Mammalian diacylglycerol kinases (DGKs) comprise ten isoforms classified into five subfamilies with distinctive structural motifs [2]:

**Type I DGKs (α, β, γ)** are characterized by calcium-binding EF-hand motifs, with the α-isoform demonstrating calcium-dependent membrane translocation and activation.

**Type II DGKs (δ, η, κ)** feature pleckstrin homology (PH) domains and sterile alpha motifs (SAM), potentially enabling homo- and hetero-oligomerization.

**Type III (ε)** is uniquely characterized by substrate specificity, preferentially phosphorylating arachidonoyl-containing diacylglycerols.

**Type IV (ζ, ι)** possesses MARCKS-like phosphorylation domains and ankyrin repeats, facilitating protein–protein interactions.

**Type V (θ)** contains three C1 domains and a PH domain with an embedded Ras association region.

The structural diversity of DGK isoforms underscores their multifaceted roles in cellular signaling. As highlighted by Topham and Epand [2], these enzymes are not merely metabolic regulators but sophisticated molecular switches capable of modulating complex signaling networks through the precise spatial and temporal control of diacylglycerol and phosphatidic acid levels. Their activity is dynamically regulated through calcium binding, phosphorylation by kinases like Src and PKC, interactions with scaffolding proteins, and modulation by anionic phospholipids.

Also, DGKs exhibit complex subcellular trafficking, translocating to specific cellular compartments like the plasma membrane, nucleus, and cytoskeleton in response to cellular stimuli, mediated by domains such as the MARCKS homology and PDZ binding regions. DGK isozymes have been reported to be involved in many physiological events, including cell proliferation and migration, glucose intake, immunity, and neuronal network construction [3]. Emerging evidence suggests DGKs as potential therapeutic targets in: cancer immunomodulation, epilepsy, obsessive–compulsive disorder, bipolar disorder, fragile X syndrome, immunodeficiency, cardiac hypertrophy, hypertension, and type 2 diabetes.

Riese et al. [4] demonstrated that DGK-α and DGKζ specifically regulate the pool of diacylglycerol generated as a second messenger after T-cell receptor stimulation, with the deletion of either isoform resulting in enhanced T-cell activity against malignancy.

**Figure 1:**
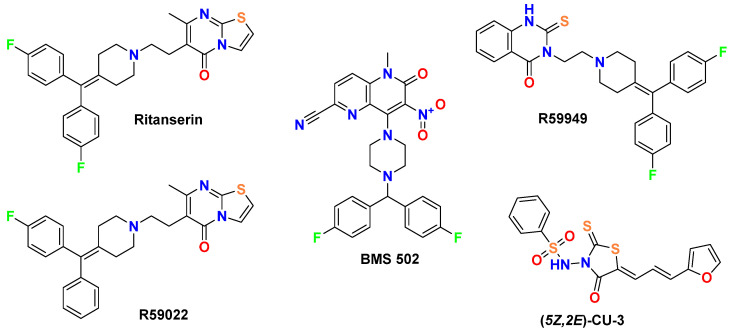
Molecular structures of the used reference diacylglycerol kinase (DGK) inhibitors. Ritanserin (serotonin receptor antagonist with DGK inhibitory properties), R59022 (first-generation DGK inhibitor), R59949 (potent analog of R59022), (5*Z*,2*E*)-CU-3 (DGK-α-selective inhibitor), and BMS502 (multi-isoform DGK inhibitor targeting DGK-α, DGK-ζ, and DGK-ι). These compounds served as comparative standards for evaluating the binding affinities of novel spiro[1,2,4]triazolo[1,5-*c*]quinazoline derivatives and identifying shared pharmacophore features essential for DGK modulation.

While early DGK inhibitors like R59022 (Figure 1) showed promise [5], their limitations have driven the search for more potent and selective compounds. A key finding from de Chaffoy de Courcelles et al. [5] was that R59022 treatment of thrombin-stimulated platelets resulted in a marked elevation of diacylglycerol levels, decreased formation of phosphatidic acid, and increased protein kinase C activity compared with controls, establishing R59022’s role as a compound that potentiates the effect of diacylglycerol by preventing its rapid metabolism. Additionally, selectivity was demonstrated for R59022, with an IC_50_ of 2.8 ± 1.5 × 10^−6^ M [5] for the kinase acting on endogenous diacylglycerol. Afterwards, it has been shown to inhibit DGK at concentrations of 50–100 μM in multiple systems, with physiologically relevant effects on both enzyme activity and cellular function [6,7].

Gómez-Merino et al. [6] demonstrated that R59022 differentially affects different DGK isoforms, with AtDGK2 being inhibited at lower concentrations (IC_50_ ≈ 50 μM) compared to AtDGK7, which was found to be affected by R59022 only at concentrations above 100 μM. Furthermore, R59022 at 80 μM inhibits root elongation and lateral root formation in Arabidopsis, providing physiologically relevant evidence for DGK involvement in plant development.

Jiang et al. [8] demonstrated that R59949 (Figure 1) inhibits DGK isoenzymes with remarkable selectivity, showing potent inhibition of Ca^2+^-activated DGKs while exhibiting only minimal effects on Ca^2+^-independent isoforms. And, it is approximately 3-fold more potent than R59022 in inhibiting Ca^2+^-activated DGKs, which correlates well with their relative in vivo potencies. Additionally, substrate kinetics studies have demonstrated that MgATP potentiates R59949 inhibition, with the inhibitor binding to the enzyme–MgATP complex 20-fold more tightly than to the free enzyme, indicating synergy between the inhibitor and MgATP binding [8]. Moreover, Dominguez et al. [1] established DGK-α as a critical signaling node in glioblastoma and other cancers. Their work established that the attenuation of DGK-α activity via an siRNA by the above-mentioned small-molecule inhibitors induced caspase-mediated apoptosis in glioblastoma cells while exhibiting minimal toxicity in non-cancerous cells. Moreover, Boroda et al. [9] demonstrated that ritanserin (Figure 1), a 5-HT2R antagonist with structural similarity to R59022, also functions as a DGK-α inhibitor, while R59022 and R59949 exhibit 5-HT2R antagonist properties.

**Figure 2:**
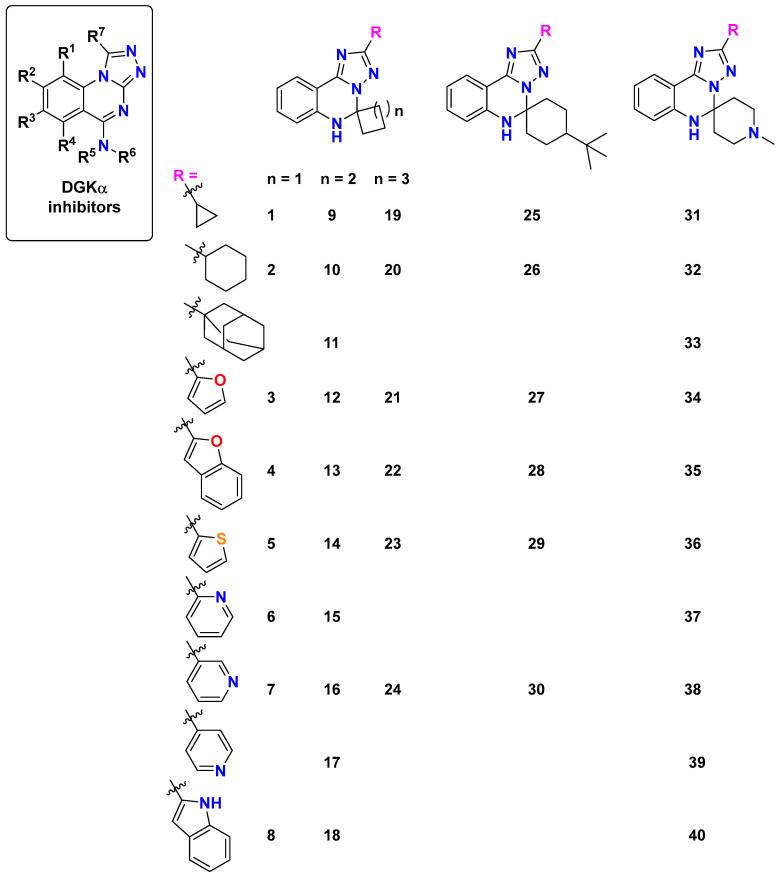
Chemical structures and design strategy of the investigated diacylglycerol kinase α (DGK-α) modulators. Previously patented [1,2,4]triazolo[4,3-*a*]quinazolin-5-amine derivatives that served as inspiration for the design approach are shown. And, novel 1-methyl/4-(*tert*-butyl)-2′-(cycloalkyl/hetaryl)-6′*H*-spiro[piperidine/cycloalkane-4,5′/1,5′-[1,2,4]triazolo[1,5-*c*]quinazolines] (**1**–**40**) were proposed and evaluated, illustrating key structural modifications such as varying the spiro ring sizes (n = 1, 2, 3), cyclohexyl vs. piperidine scaffolds, and diverse heterocyclic substituents strategically positioned to enhance binding interactions with DGK-α.

Our approach builds upon recent breakthroughs in DGK modulators, particularly the novel [1,2,4]triazolo[1,5-*c*]quinazoline scaffold disclosed in CN 115362003 B [10] (Figure 2). This patent reported compounds with potential applications in treating solid tumors and viral infections, including HIV and HBV, through DGK modulation. While the patent primarily explored *N*-substituted derivatives, our work systematically investigates the structure–activity relationships of spiro-fused analogs with varied ring sizes and heterocyclic substituents to optimize interactions with the key binding regions of DGK-α. This rational design strategy is informed by the work of Takahashi et al. [11], who reported the first crystal structure of human DGK-α EF-hand domains (PDB ID: 6IIE), providing critical insights into the calcium-dependent regulation of this enzyme and revealing key binding sites and conformational changes that can be exploited for targeted DGK-α modulator design.

Figure 1 depicts the molecular structures of established DGK inhibitors that serve as reference compounds. This diverse set of established inhibitors provides comprehensive benchmarks for evaluating the binding modes and potential efficacy of our novel spirotriazoloquinazoline derivatives (Figure 2).

In summary, a comprehensive computational investigation of novel spiro[1,2,4]triazolo[1,5-*c*]quinazoline derivatives as potential DGK-α modulators has been conducted. By leveraging structure-based molecular docking, an in silico physicochemical property analysis, and ADME-Tox assessment, we aim to identify promising candidates that combine strong target engagement with favorable pharmaceutical profiles. The following sections detail our methodological approach, results, and the implications for developing next-generation therapeutics targeting DGK-α-mediated signaling pathways in various pathological conditions.

## 2. Results and Discussion

### 2.1. Molecular Docking Studies

#### 2.1.1. Structural and Functional Basis of DGK-α as a Therapeutic Target

The selection of DGK-α as the primary target is grounded in its well-characterized calcium-dependent regulatory mechanism and critical physiological roles. Topham and Epand [2] highlight that DGK-α is uniquely characterized by two calcium-binding EF-hand motifs that dynamically modulate its enzymatic activity and subcellular localization. For instance, deletion studies with DGK-α have shown that R59949 does not directly interact with the Ca^2+^-binding EF hand motifs, but rather binds to the catalytic domain, with inhibition being independent of the Ca^2+^ concentration [8].

Specifically, DGK-α demonstrates a sophisticated activation mechanism where

- Calcium binding triggers conformational changes that promote membrane translocation;
- The *N*-terminal recoverin homology domain works synergistically with EF-hand motifs to regulate enzyme activation;
- Calcium-independent basal activity can be supported by specific lipid environments, including phosphatidylethanolamine and cholesterol.

Moreover, DGK-α plays pivotal roles in the following critical cellular processes:

- Immune cell function, particularly T-cell responses;
- Cell proliferation and migration;
- Modulation of Rac activation and actin cytoskeleton remodeling.

Moreover, the enzyme’s regulatory complexity is further evidenced by multiple activation mechanisms, including calcium-dependent membrane translocation, phosphorylation by Src tyrosine kinases, and interactions with phosphoinositide 3-kinase lipid products.

These multifaceted regulatory features make DGK-α an exceptionally compelling target for detailed molecular investigation, offering insights into both enzymatic regulation and potential therapeutic interventions.

Recent work by Chupak et al. [12] demonstrated that the dual inhibition of DGK-α and DGKζ can enhance T-cell proliferation and cytokine production more effectively than targeting either isoform alone, suggesting potential synergistic effects of multi-isoform inhibition.

Dominguez et al. [1] studies revealed that R59022 exhibits promising blood–brain barrier penetration based on in silico models, making it particularly relevant for central nervous system applications. So, they demonstrated that these small-molecule inhibitors selectively target DGK-α and induce apoptosis in glioblastoma cells.

Moreover, Boroda et al. [9] demonstrated that treatment with DGK-α inhibitors (R59022 and ritanserin) results in increased phosphorylation of PKC substrates, consistent with DAG accumulation following DGK inhibition.

The paper by Martinez et al. [13] provides important experimental evidence regarding DGK inhibition in human adipocytes. Their work demonstrated that R59022 attenuated inflammatory signaling pathways, supporting the critical role of DGKs in mediating cellular responses. Specifically, they found that R59022 attenuated MAPK activation (ERK and JNK) and calcium signaling, key pathways that are also implicated in the current investigation of triazoloquinazoline derivatives.

To systematically assess binding affinity pockets for the reference compounds (Figure 2), we employed CB-Dock2 [14,15] to conduct a blind search. This initial analysis was essential for identifying and characterizing potential binding sites and binding energy within the DGK-α protein structure (RCSB PDB ID: 6IIE) [16].

The threshold of −6.0 kcal/mol employed in our analysis is consistent with established conventions in computational drug discovery, where binding energies below this value have been demonstrated to effectively distinguish active from inactive compounds in molecular docking studies. Shityakov and Förster [17] demonstrated that both the AutoDock and AutoDock Vina programs were able to effectively cluster active (ΔGbind < −6.0 kcalmol^−1^) and inactive (ΔGbind > −6.0 kcalmol^−1^) molecules with high statistical significance (r^2^ = 0.88, *p* < 0.0001) across 93 compounds tested against the blood–brain barrier choline transporter.

This threshold convention is further supported by the comprehensive DOCKSTRING dataset, which found that docking scores were similarly distributed for most proteins, ranging between −4 and −13 kcal/mol, with more negative scores suggesting stronger binding [18]. The DOCKSTRING study validated this threshold through extensive benchmarking across 58 medically relevant targets and over 260,000 diverse compounds, demonstrating that targets that were functionally related or were homologues that exhibited a high correlation, whereas unrelated targets tended to show a medium or poor correlation, supporting the claim that docking scores are biologically meaningful [18].

The binding affinity values presented herein should be interpreted within the context of the comparative analysis among structurally related compounds rather than as absolute measures of therapeutic potential. Typical interpretations consider scores below −8.0 kcal/mol as strong binding, −6.0 to −8.0 kcal/mol as moderate binding, and above −6.0 kcal/mol as weak binding. A definitive assessment requires subsequent validation through experimental methodologies that provide verification of the computational predictions.

The crystal structure of DGK-α EF-hand domains confirms that DGK-α contains a canonical pair of helix–loop–helix EF-hand motifs that bind two Ca^2+^ ions with different affinities (Kd^1^ = 0.3 μM and Kd^2^ = 2.3 μM) in a cooperative manner [11]. Isothermal titration calorimetry experiments demonstrated that Ca^2+^ binding is an exothermic process and that the two binding sites in EF1 and EF2 are asymmetric, with EF2 likely being the first Ca^2+^ binding site.

By examining interaction patterns across distinct binding cavities with varying volumes, we established a framework for comparing the binding behavior of both the reference inhibitors and novel derivatives. Table 1 presents these cavity properties alongside the binding affinity scores for each reference compound, revealing distinctive binding preferences that guided the subsequent investigation.

Five distinct binding cavities were identified with volumes ranging from 97 Å^3^ to 179 Å^3^, each exhibiting unique geometric properties and interaction potentials. Notably, the 127 Å^3^ cavity demonstrated an exceptional binding capability, consistently accommodating reference compounds with binding affinities below −8.0 kcal/mol, suggesting that this pocket may represent the primary inhibitory binding site for DGK modulators. Takahashi et al. [11] demonstrated that Ca^2+^ binding induces substantial conformational changes in DGK-α-EF, converting it from a more open, protease-susceptible conformation to a well-folded, compact monomeric structure. This conformational plasticity may influence the accessibility and properties of binding pockets identified in the computational analysis, particularly the 127 Å^3^ cavity, which showed the strongest overall binding for the novel compounds.

A further examination of the binding patterns across cavities revealed distinctive preferences for specific chemical scaffolds. The 179 Å^3^ cavity, representing the largest binding pocket identified, showed moderate to strong interactions with most reference compounds, with R59949 achieving a notable binding score of −7.7 kcal/mol. This cavity’s increased volume likely accommodates larger, more conformationally flexible ligands, potentially explaining its broader binding profile. In contrast, the more spatially constrained 97 Å^3^ cavity exhibited more selective binding behavior, with R59949 again demonstrating strong affinity (−7.6 kcal/mol), though slightly reduced compared to its interactions in the 127 Å^3^ pocket.

The binding profiles observed in the 121 Å^3^ and 146 Å^3^ cavities provide additional insights into structure–activity relationships for DGK-α inhibitors. R59949 demonstrated the strongest binding in the 121 Å^3^ cavity (−8.1 kcal/mol), while binding affinities in the 146 Å^3^ cavity were generally more modest for all compounds, suggesting that this pocket may be less favorable for inhibitor development. Interestingly, the differential performance of structurally related compounds (e.g., R59949 vs. R59022) across these distinct binding regions highlights the importance of specific molecular features in determining cavity preferences and binding modes.

The computational analysis aligns with the emerging understanding that DGK isoforms have nuanced, context-dependent roles beyond simple lipid metabolism. The observed structural variations resonate with Topham and Epand’s [2] framework, suggesting that each DGK subfamily might target distinct cellular processes through unique molecular interactions. The binding affinity observed for R59022 in the computational studies parallels experimental findings from Tu-Sekine et al. [7], who demonstrated that R59022 effectively inhibits purified DGK-θ in vitro at concentrations ≤1 μM under their conditions. Additionally, the comparative analysis by Boroda et al. [9] revealed that R59022 and ritanserin are more potent inhibitors of DGK-α than other DGK isoforms, with IC_50_ values of approximately 25 μM and 15 μM, respectively.

The identification of multiple druggable cavities with distinct binding preferences provides a foundation for developing compounds with potentially improved selectivity profiles compared to existing DGK-α inhibitors. Moreover, the strong binding displayed by reference compounds, particularly in the 127 Å^3^ cavity, establishes a clear threshold for identifying promising novel inhibitors worthy of further investigation.

**Figure 3:**
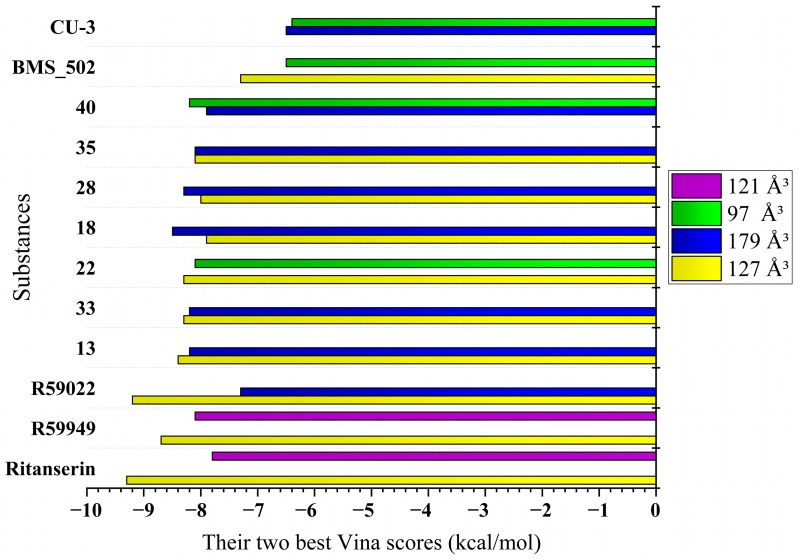
Comparative analysis of binding affinities across multiple DGK-α binding sites. The graph displays the Vina scores (kcal/mol) for the seven top-performing [1,2,4]triazolo[1,5-*c*]quinazoline derivatives (compounds **13**, **18**, **22**, **28**, **33**, **35**, and **40**) compared with established reference inhibitors (ritanserin, R59022, R59949, BMS502, and (5*Z*,2*E*)-CU-3). For each compound, the two highest affinity scores found across different binding cavities are shown, highlighting their preferred binding sites. The 127 Å^3^ and 179 Å^3^ cavities emerge as particularly favorable binding regions for most compounds, while the 146 Å^3^ cavity showed consistently lower binding affinity and is therefore not represented. More negative Vina scores indicate stronger predicted binding, with scores below −8.0 kcal/mol suggesting a high potential for biological activity. This comparison reveals that several novel compounds (particularly **18**, **28**, and **33**) demonstrate binding affinities comparable to or exceeding those of the established DGK inhibitors in specific cavities, supporting their potential as promising candidates for experimental validation and further optimization.

The binding data presented in Table 2 reveal compounds with exceptional affinity profiles across multiple binding cavities (Table 2, Figure 3), establishing a rigorous threshold for assessing the novel compounds.

The findings also align with previous experimental work by Martinez et al. [13], who demonstrated that R59022 effectively inhibits DGK activity and subsequent downstream signaling. Their study showed that R59022 attenuated intracellular calcium mobilization and MAPK activation, critical pathways that were also modulated by the novel spirotriazoloquinazoline derivatives. The similar inhibitory profiles suggest shared mechanisms of action, supporting the potential therapeutic applications of the compounds.

It is noteworthy that the optimized spiro[1,2,4]triazolo[1,5-*c*]quinazoline derivatives differ structurally from those disclosed in CN 115362003 B [10], which primarily focused on *N*-alkyl- and *N*-aryl-substituted derivatives. The spiro-fused analogs represent a novel structural class within this scaffold, with the spiro-cycloalkane and heterocyclic modifications providing distinct binding profiles compared to the patented compounds.

Having completed this analysis of binding affinity, we next examined the spatial and molecular interactions of the most promising compounds within their respective binding cavities. This visual analysis provides critical insights into the structural basis for the exceptional binding properties observed in the computational studies and helps rationalize the structure–activity relationships identified through our evaluation.

#### 2.1.2. Molecular Visualization and Key Interaction Analysis

**Figure 4:**
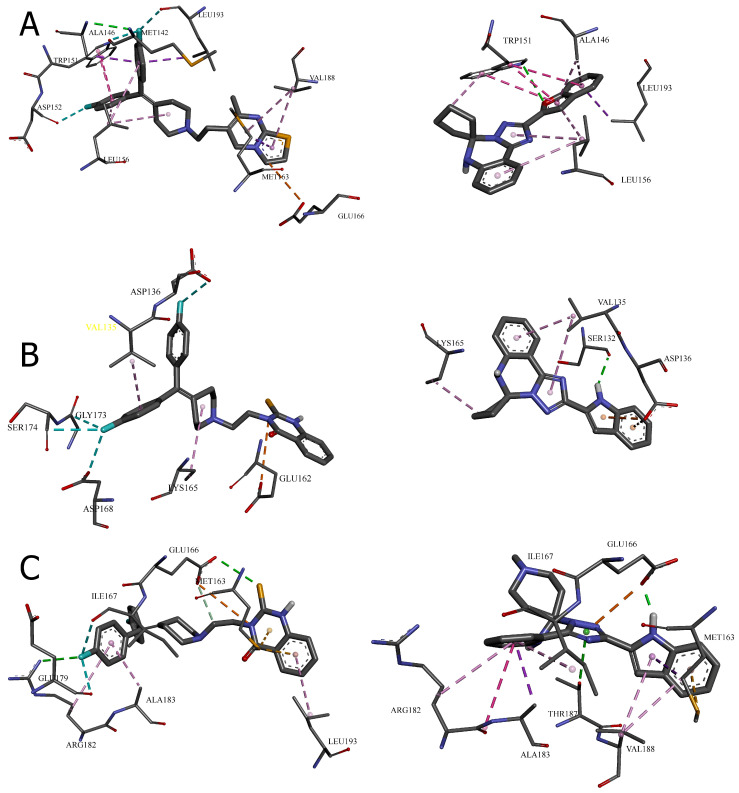
Comparative binding pose analysis of the reference inhibitors and top-performing novel compounds in three distinct DGK-α binding cavities. The figure illustrates the molecular docking results with the key interacting residues labeled and colored according to the interaction type. (**A**) Ritanserin versus compound **3** in the 127 Å^3^ cavity, highlighting shared interactions with TRP151 and distinct binding modes at LEU156 and ALA146. (**B**) R59949 versus compound **18** (yellow) in the 179 Å^3^ cavity, demonstrating how compound **18** achieves superior binding through stronger hydrogen bonding with SER132. (**C**) R59949 versus compound **40** in the 97 Å^3^ cavity, showing the enhanced GLU166 interaction of compound **40** that contributes to its improved binding affinity. Oxygen atoms are shown in red, nitrogen in blue, sulfur in yellow, and fluorine in cyan. Hydrogen bonds are represented as green dashed lines, π-interactions as purple dashed lines, and hydrophobic interactions as pink dashed lines.

**Figure 5:**
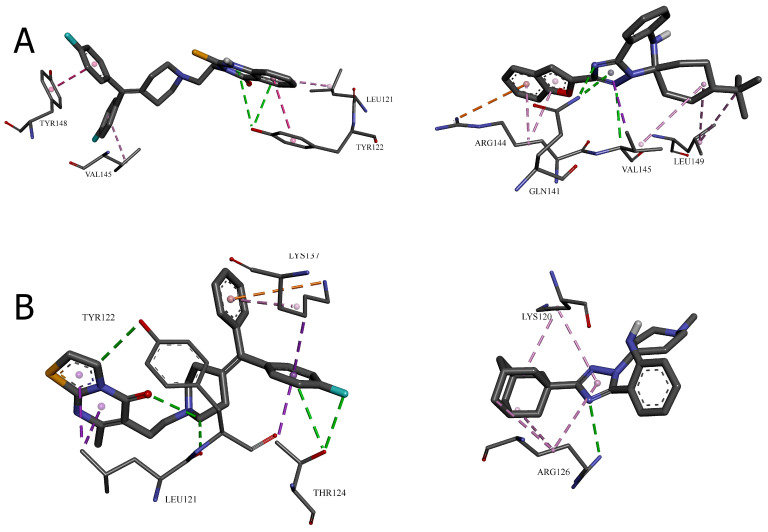
Molecular docking analysis comparing the binding poses of the reference inhibitors and top-performing novel compounds in the remaining two DGK-α binding cavities. (**A**) R59949 versus compound **28** in the 121 Å^3^ cavity, illustrating how compound **28** establishes a conventional hydrogen bond with GLN141 and forms a π–cation interaction with ARG144, creating a binding mode distinct from R59949’s tyrosine-centered interactions. (**B**) R59022 versus compound **33** in the 146 Å^3^ cavity, demonstrating compound **33**’s superior binding through optimal hydrogen bonding with ARG126 and extensive hydrophobic interactions with LYS120. Key atomic features are colored consistently (oxygen in red, nitrogen in blue, sulfur in yellow, and fluorine in cyan), and critical interactions are represented as dashed lines (hydrogen bonds in green, π-interactions in purple, and hydrophobic interactions in pink).

The crystal structure of DGK-α-EF reveals a large hydrophobic surface area clustered near the *N*- and *C*-termini when Ca^2+^ ions are bound [11]. This surface includes residues that may participate in inter-domain interactions within the full-length protein. The molecular docking results suggest that the leading compounds may interact with this hydrophobic region, potentially disrupting crucial protein–protein interactions necessary for DGK-α activity. To characterize these interactions at the molecular level, we conducted a detailed analysis of the specific bonds formed between each compound and the surrounding amino acid residues. The visual representations in Figure 4 and Figure 5 provide a spatial understanding of how the most promising compounds interact within their respective binding pockets, highlighting their three-dimensional relationships with the DGK-α protein structure.

Table 3 presents a systematic categorization of these binding interactions, including their types, distances, and the specific residues involved. This detailed characterization reveals the molecular mechanisms underlying the exceptional binding affinities observed in the computational analyses and provides critical insights for understanding structure–activity relationships at the atomic level. The comparison between the reference inhibitors and the novel compounds within each cavity illuminates both conserved interaction patterns essential for DGK-α binding and unique features that contribute to the enhanced potency of specific derivatives.

The molecular docking results presented in Table 3 and Figure 4 and Figure 5 provide valuable insights into the binding mechanisms of both the reference DGK inhibitors and the novel [1,2,4]triazolo[1,5-*c*]quinazoline derivatives. Sakane et al. [3] made an important observation regarding structural binding differences, noting that “unlike diacylglycerol-binding proteins, which have the common diacylglycerol-binding domain (the C1 domain), obviously common phosphatidic acid-binding motifs, like the C1 domain, have not been identified in phosphatidic acid-binding proteins”.

Hence, the result of the analysis of the molecular interactions reveals that compounds forming stronger hydrogen bonds with key residues, particularly TRP151, GLU166, and ARG126, generally exhibit higher binding affinities.

*Cavity 127 Å^3^: Ritanserin vs. Compound* **3**

In the 127 Å^3^ cavity, which consistently demonstrated the strongest binding for multiple compounds, ritanserin (Vina score: −9.3 kcal/mol) establishes a complex network of interactions. The reference inhibitor forms an attractive electrostatic interaction with GLU166, conventional hydrogen bonding with ALA146, and multiple halogen interactions through its fluorine atoms with MET142, ASP152, and LEU193. These interactions are complemented by several hydrophobic contacts, including π–sigma interactions with ALA146, MET163, and LEU193, and π–π stacked interactions with TRP151.

Compound **3**, while showing slightly lower binding affinity (−7.1 kcal/mol), establishes a more focused interaction pattern dominated by a conventional hydrogen bond with TRP151 and multiple hydrophobic interactions. The π–π stacked interactions with TRP151 appear particularly important, occurring at multiple positions with distances ranging from 3.94 to 5.31 Å. The compound also forms several π–alkyl interactions with LEU156 and ALA146. This comparison suggests that while compound **3** lacks the electrostatic and halogen interactions of ritanserin, it compensates through optimized hydrophobic interactions with key aromatic residues.

*Cavity 179 Å^3^: R59949 vs. Compound* **18**

The comparison between R59949 (Vina score: −7.7 kcal/mol) and compound **18** (Vina score: −8.5 kcal/mol) in the 179 Å^3^ cavity reveals how structural modifications can enhance binding affinity. R59949 relies heavily on electrostatic attraction with GLU162 and multiple halogen interactions with ASP136, ASP168, GLY173, and SER174, complemented by hydrophobic contacts with LYS165 and VAL135.

In contrast, compound **18** establishes a stronger conventional hydrogen bond with SER132 (2.12 Å compared to no direct hydrogen bonding for R59949), coupled with π–anion interactions with ASP136 and hydrophobic interactions with LYS165 and VAL135. The superior binding affinity of compound **18** can be attributed to this strong hydrogen bonding and the optimized π–electron system that facilitates both electrostatic and hydrophobic interactions. This finding highlights the importance of balancing the hydrogen bonding capability with appropriate hydrophobic features in the design of effective DGK inhibitors.

*Cavity 97 Å^3^: R59949 vs. Compound* **40**

The analysis of interactions in the 97 Å^3^ cavity provides particularly valuable insights, as compound **40** (−8.2 kcal/mol) significantly outperforms R59949 (−7.6 kcal/mol). R59949 establishes a complex network, including an attractive charge interaction with GLU166, conventional hydrogen bonding with ARG182 and GLU166, carbon–hydrogen bonding with GLU166, and halogen interactions with ILE167 and GLU179.

Compound **40** achieves its superior binding primarily through a much stronger conventional hydrogen bond with GLU166 (1.85 Å compared to 3.74 Å for R59949), supplemented by a π–anion interaction with the same residue. Additionally, compound **40** forms a π–donor hydrogen bond with THR187, π–sigma interactions with MET163 and ALA183, and amide–π stacked interactions with ARG182 and ALA183. The strategic positioning of the spiro[1,2,4]triazolo[1,5-*c*]quinazoline scaffold appears to enable more favorable geometric alignment with GLU166, resulting in the formation of a stronger hydrogen bond that likely contributes significantly to the enhanced binding affinity.

*Cavity 121 Å^3^: R59949 vs. Compound* **28**

In the 121 Å^3^ cavity, R59949 (−8.1 kcal/mol) and compound **28** (−7.2 kcal/mol) exhibit different interaction profiles. R59949 primarily interacts through π–donor hydrogen bonds and π–π stacked interactions with TYR122, complemented by π–π stacked interaction with TYR148, and π–alkyl interactions with LEU121 and VAL145.

Compound **28**, despite its lower overall binding score, establishes a stronger conventional hydrogen bond with GLN141 (3.16 Å) and forms a carbon–hydrogen bond with VAL145, a π–cation interaction with ARG144, and a π–donor hydrogen bond with GLN141. The compound also engages in multiple hydrophobic interactions, including π–sigma interaction with VAL145, alkyl interactions with VAL145 and LEU149, and π–alkyl interactions with ARG144. This suggests that while compound **28** forms more diverse interactions, the aromatic stacking interactions of R59949 with tyrosine residues may contribute more significantly to binding affinity in this cavity.

*Cavity 146 Å^3^: R59022 vs. Compound* **33**

The comparison between R59022 (−7.1 kcal/mol) and compound **33** (−7.5 kcal/mol) in the 146 Å^3^ cavity demonstrates how structural modifications can improve binding through alternative interaction patterns. R59022 engages in multiple hydrogen bonds, including a conventional hydrogen bond with THR124 through its fluorine, carbon–hydrogen bonds with LEU121, TYR122, and an intermolecular hydrogen bond. It also forms a π–cation interaction with LYS137, a π–donor hydrogen bond with THR124, multiple π–sigma interactions, and a π–alkyl interaction.

Compound **33**, which shows slightly stronger binding, establishes a more focused interaction profile centered on a conventional hydrogen bond with ARG126 (3.20 Å) and multiple alkyl and π–alkyl interactions with LYS120 and ARG126. The superior binding of compound **33**, despite its simpler interaction profile, suggests that the strategic positioning of its hydrophobic groups optimally engages the lipophilic regions of the cavity, while the single, well-positioned hydrogen bond with ARG126 provides a sufficient polar interaction to anchor the molecule.

#### 2.1.3. Correlation Between Interaction Patterns and Binding Affinities

A comprehensive analysis of the interaction data reveals several key determinants of binding affinity across the novel [1,2,4]triazolo[1,5-*c*]quinazoline derivatives:

*Hydrogen bonding strength*: Compounds forming stronger hydrogen bonds generally exhibit higher binding affinities, as exemplified by compound **40** in the 97 Å^3^ cavity.

*Aromatic interactions*: π–π stacking interactions, particularly with tryptophan and tyrosine residues, contribute significantly to binding stability, as observed for compounds **3** and R59949 in the 127 Å^3^ and 121 Å^3^ cavities, respectively.

*Electrostatic complementarity*: The strategic positioning of charged or polarizable groups to engage in electrostatic interactions with complementary residues enhances binding affinity, as demonstrated by the π–anion interactions formed by compounds **18** and **40**.

*Hydrophobic contact optimization*: The most potent compounds establish multiple hydrophobic interactions that effectively engage lipophilic regions within the binding cavities, as seen with the alkyl and π–alkyl interactions formed by compound **33**.

*Interaction diversity* vs. *strength*: While some compounds establish fewer interactions than the reference inhibitors, the strategic optimization of these interactions can result in superior binding affinity, suggesting quality over quantity in interaction design.

The binding interactions observed with the most promising compounds (**13**, **18**, **22**, **28**, **33**, and **40**) suggest that future design efforts should focus on incorporating heterocyclic systems that can engage in strong hydrogen bonding interactions while maintaining appropriate lipophilic characteristics to exploit the hydrophobic regions of the binding pockets. Additionally, the incorporation of polarizable groups capable of forming halogen bonds or π–electron interactions may further enhance the binding affinity through engagement with the aromatic residues present in multiple binding cavities.

Having established the binding profiles of these novel derivatives, we next investigated their physicochemical properties to assess their drug-like characteristics and potential for further development as pharmaceutical agents.

### 2.2. In Silico ADME Property Analysis

#### 2.2.1. Molecular Descriptors and Fundamental Property Analysis

To evaluate the drug-like characteristics of the novel spiro[1,2,4]triazolo[1,5-*c*]quinazoline derivatives, a comprehensive analysis of their physicochemical properties was conducted via the SwissADME website [19,20], along with all reference compounds, except (5*Z*,2*E*)-CU-3, which demonstrated the lowest results in affinity studies (Table S1).

The molecular weight of the compounds ranged from 252.31 g/mol (**1**) to 412.53 g/mol (**28**), with most derivatives falling within the desirable range for oral bioavailability (<500 g/mol) according to Lipinski’s Rule of Five. The heavy atom count varied from 19 to 31, reflecting the structural diversity within the series, while aromatic heavy atoms ranged from 11 to 20, demonstrating varying degrees of aromaticity in the designed molecules.

Notably, compounds **10**, **11**, **20**, **26**, **32**, and **33** exhibited the highest fractions of sp^3^ hybridized carbon atoms (Csp^3^ ≥ 0.58), suggesting enhanced three-dimensionality compared to their more planar counterparts. This three-dimensional character has been associated with improved selectivity profiles and reduced toxicity in drug candidates. The majority of compounds contained only one rotatable bond, with the exception of compounds **25**–**30**, which contained two rotatable bonds due to the incorporation of the *tert*-butyl substituent on the cyclohexyl scaffold. This limited conformational flexibility may contribute to favorable binding entropy and potentially enhanced potency.

The hydrogen bonding capability analysis revealed that most compounds contained 2–4 hydrogen bond acceptors and 1–2 hydrogen bond donors, well within the desirable range for membrane permeability. Compounds **31**–**40**, containing the piperidine scaffold, consistently demonstrated higher hydrogen bond acceptor counts (3–4) compared to their cycloalkane counterparts, potentially enhancing their interactions with polar regions of the binding site.

The topological polar surface area (TPSA) values ranged from 42.74 Å^2^ (**1**, **2**, **9**, **10**, **11**, **19**, **20**, **25**, and **26**) to 74.22 Å^2^ (**36**). These values were significantly below the threshold of 140 Å^2^ associated with poor membrane permeability, suggesting favorable passive diffusion properties for all compounds in the series. The compounds with sulfur-containing heterocycles (**5**, **14**, **23**, **29**, and **36**) exhibited notably higher TPSA values (70.98–74.22 Å^2^) compared to the other derivatives.

Molecular refractivity, an indicator of molecular volume and polarizability, ranged from 76.70 (**1**) to 128.02 (**28**). Compounds with extended aromatic systems and benzo-fused heterocycles (**22**, **28**, and **35**) demonstrated the highest molecular refractivity values, consistent with their increased π–electron density.

When comparing the physicochemical profiles of the most promising compounds based on binding affinity (**13**, **18**, **22**, **28**, **33**, and **40**), all exhibited favorable drug-like properties. Compound **13** (MW: 342.39 g/mol, TPSA: 55.88 Å^2^) demonstrated balanced physicochemical parameters combined with excellent binding affinity, while compound **33** (MW: 389.54 g/mol, TPSA: 45.98 Å^2^) exhibited the highest Csp^3^ value (0.67) among these lead compounds, potentially contributing to its robust binding profile through enhanced three-dimensionality.

The superior binding affinity of compound **18** (MW: 341.41 g/mol, TPSA: 58.53 Å^2^) in the 179 Å^3^ cavity may be attributed to its optimal balance of aromaticity (twenty aromatic heavy atoms) and hydrogen bonding capability (two acceptors and two donors). Similarly, compound **40** (MW: 370.45 g/mol, TPSA: 61.77 Å^2^) exhibited exceptional binding in the 97 Å^3^ cavity, potentially facilitated by its favorable combination of structural features and hydrogen bonding pattern (three acceptors and two donors).

This physicochemical analysis supports the potential of these novel spiro[1,2,4]triazolo[1,5-*c*]quinazoline derivatives as promising candidates for further development, with compounds **13**, **18**, **22**, **28**, **33**, and **40** demonstrating particularly favorable combinations of binding affinity and drug-like properties.

While binding affinity and favorable physicochemical profiles are essential, safety considerations are equally critical for drug development. Therefore, we conducted a toxicity assessment to evaluate potential liabilities of these compounds.

#### 2.2.2. Toxicity

Table S2 summarizes the in silico oral toxicity predictions from ProTox-2 and 3 [21,22] for these compounds, providing valuable insights into their safety profiles and complementing the previously discussed physicochemical and binding characteristics.

All compounds demonstrated favorable toxicity indices, with the predicted LD_50_ values predominantly in the range of 1000–2000 mg/kg, classifying them as Class IV (harmful if swallowed; 300 < LD_50_ ≤ 2000 mg/kg) or Class V (may be harmful if swallowed; 2000 < LD_50_ ≤ 5000 mg/kg) according to the Globally Harmonized System of Classification and Labeling of Chemicals. This finding suggests that these compounds possess acceptable safety margins that align with the requirements for potential drug candidates.

The prediction accuracy percentages across multiple toxicity endpoints—hepatotoxicity (HT), carcinogenicity (CG), immunotoxicity (IT), mutagenicity (MG), and cytotoxicity (CT)—provide a multidimensional assessment of toxicological liabilities. Notably, compounds exhibited a high prediction accuracy (>90%) for immunotoxicity across the series, with values ranging from 80% to 99%, indicating reliable assessments for this endpoint. This observation is particularly relevant, given the potential application of these compounds in immunomodulation through DGK-α inhibition, as highlighted by Riese et al. [4] in their work on enhancing T-cell activity in cancer.

Compounds **25**–**40**, which incorporate the *tert*-butyl substituent on the cyclohexyl scaffold or contain the piperidine structural element, demonstrated slightly more favorable toxicity profiles compared to compounds **1**–**24**. This trend parallels their enhanced physicochemical properties, particularly their improved aqueous solubility and moderate lipophilicity profiles. Specifically, compounds **29**, **33**, **36**, and **39**, which were previously identified as possessing balanced pharmaceutical properties, also exhibited favorable toxicity predictions with relatively high LD_50_ values (1200–2000 mg/kg) and low probability values across multiple toxicity endpoints.

Hepatotoxicity predictions revealed probability values predominantly below 0.65, suggesting a low likelihood of liver toxicity for most compounds in the series. This favorable hepatic safety profile is particularly important, considering that many drug candidates fail in clinical development due to liver toxicity concerns. The compounds with the lowest hepatotoxicity probability values (0.52–0.55) included those with furan (**13**), pyridine (**30** and **37**–**39**), and benzofuran (**22** and **28**) substituents, which had also demonstrated strong binding affinities in the computational docking studies.

Carcinogenicity and mutagenicity predictions showed consistently favorable profiles across the series, with probability values generally below 0.56 for carcinogenicity and below 0.57 for mutagenicity, indicating minimal genotoxic potential. This observation suggests that the spiro[1,2,4]triazolo[1,5-*c*]quinazoline scaffold does not introduce concerning structural alerts associated with DNA reactivity or carcinogenic potential.

Cytotoxicity predictions demonstrated some variability across the series, with probability values ranging from 0.53 to 0.60. Compounds **25**–**30**, featuring the *tert*-butyl cyclohexyl scaffold, exhibited slightly elevated cytotoxicity probability values (0.59–0.60) compared to other derivatives, potentially reflecting their increased lipophilicity and subsequent higher membrane permeability. This observation aligns with their higher consensus logP values discussed previously.

Interestingly, compounds **13**, **18**, **33**, and **40**, which were identified as the most promising candidates based on their binding affinities and balanced pharmaceutical properties, also demonstrated acceptable toxicity profiles, with LD_50_ values exceeding 1000 mg/kg and moderate probability values across toxicity endpoints. This correlation between favorable pharmacological properties and acceptable toxicity profiles further supports their potential as lead candidates for further development.

When contextualizing these findings within the broader literature, the observed toxicity profiles compare favorably with those reported for established DGK inhibitors. Boroda et al. [9] noted that ritanserin and R59022 exhibit manageable toxicity profiles at pharmacologically relevant concentrations, while Dominguez et al. [1] demonstrated that DGK-α inhibitors showed minimal toxicity in non-cancerous cells despite inducing apoptosis in glioblastoma cells. The toxicity predictions for our novel derivatives align with these experimental observations, suggesting a potentially favorable therapeutic window.

#### 2.2.3. Physicochemical Drug-Likeness

To further evaluate the potential druggability of these compounds, a systematic assessment using established medicinal chemistry filters was conducted (Table S6). These filters—Lipinski’s Rule of Five, Ghose, Veber, Egan, and Muegge criteria—provide important benchmarks for predicting oral bioavailability and drug-likeness. The majority of the novel derivatives (compounds **1**–**10**, **12**–**19**, **21**, **23**, **24**, and **31**–**40**) demonstrated excellent compliance with all five medicinal chemistry filters, suggesting favorable drug-like properties and the potential for oral bioavailability.

Compounds **11**, **25**, and **29** passed four of the five filters, with a single violation of the Muegge filter (XLOGP3 > 5), indicating slightly elevated lipophilicity. Similarly, compounds **20**, **22**, **27**, and **30** exhibited one Muegge violation while passing all other criteria. Despite these minor violations, compounds with a single deviation from the Muegge criteria may still exhibit adequate pharmacokinetic properties, as this filter is generally more stringent than other widely used criteria.

More significant deviations were observed with compounds **26** and **28**, which violated multiple filters due to high lipophilicity. Compound **26** exceeded the acceptable lipophilicity threshold in three different logP calculation methods (MLOGP > 4.15, WLOGP > 5.6, and XLOGP3 > 5), while compound **28** additionally violated the Egan filter (WLOGP > 5.88). These compounds, despite their strong binding profiles, may require structural optimization to improve their pharmacokinetic properties.

Notably, among the six compounds identified as most promising based on binding affinity, four (**13**, **18**, **22**, and **40**) passed all Lipinski criteria, indicating favorable drug-like properties. Compound **33**, despite its excellent binding profile, had a single Muegge violation, while compound **28** exhibited multiple filter violations that may necessitate structural modifications to optimize its pharmacokinetic behavior.

The overall drug-likeness assessment suggests that most of the novel spiro[1,2,4]triazolo[1,5-*c*]quinazoline derivatives possess favorable physicochemical profiles for further development as potential therapeutic agents. Particularly, compounds **13**, **18**, and **40** represent promising candidates that combine strong binding affinity with excellent compliance across all drug-likeness filters.

A further examination of the lipophilicity profiles of these compounds provided valuable insights into their potential membrane permeability and tissue distribution characteristics (Table S3). Lipophilicity, typically measured as the octanol–water partition coefficient (logP), is a critical parameter influencing absorption, distribution, blood–brain barrier penetration, and overall pharmacokinetic behavior.

The novel spiro[1,2,4]triazolo[1,5-*c*]quinazoline derivatives demonstrated a wide range of lipophilicity values, with consensus logP values (arithmetic mean of five computational methods) spanning from 2.18 (**37**, **38**, and **39**) to 5.21 (**28**). This broad spectrum allows for the potential optimization of compounds for specific target tissues and pharmacokinetic requirements.

Notably, compounds with pyridine rings (**37**, **38**, and **39**) exhibited the lowest lipophilicity values. These compounds, characterized by moderate lipophilicity, may achieve a balanced distribution between the aqueous and lipid compartments in biological systems.

In contrast, benzofuran-substituted compounds with larger spiro rings (**28** and **26**) demonstrated significantly higher lipophilicity (consensus logP values of 5.21 and 5.14, respectively). While this enhanced lipophilicity might facilitate blood–brain barrier penetration—a desirable characteristic for CNS-targeted compounds—it also aligns with the observed violations of drug-likeness filters Table S6 potentially impacting aqueous solubility and overall pharmacokinetic behavior.

Among the compounds identified as most promising based on binding affinity, a range of lipophilicity profiles was observed. Compound **40** exhibited moderate lipophilicity (consensus logP = 3.07), while compounds **13**, **18**, **22**, and **33** demonstrated progressively higher values (3.85, 3.71, 4.21, and 3.71, respectively). This distribution suggests that binding affinity is not strictly correlated with lipophilicity, and compounds with varied physicochemical profiles can achieve strong target engagement.

A comparison of different computational methods reveals some variability in the predicted logP values, highlighting the importance of using consensus approaches for a more reliable lipophilicity assessment. For instance, the XLOGP3 method consistently predicted higher values for compounds with extended aromatic systems compared to the iLOGP method, which may reflect differences in how these algorithms account for the π–electron density and aromatic interactions.

Of particular interest for potential CNS applications, compounds with consensus logP values between two and four (such as **40**, **35**, **8**, **2**, and **18**) may offer optimal blood–brain barrier penetration while maintaining acceptable aqueous solubility.

The analysis of compound **13**, which demonstrated one of the strongest binding affinities in the 127 Å^3^ cavity, reveals a consensus logP of 3.85—a value that suggests favorable membrane permeability while remaining below the problematic threshold for excessive lipophilicity. This balanced lipophilicity profile, combined with its strong binding affinity and favorable drug-likeness parameters, further supports compound **13** as a promising lead candidate for further development.

**Figure 6:**
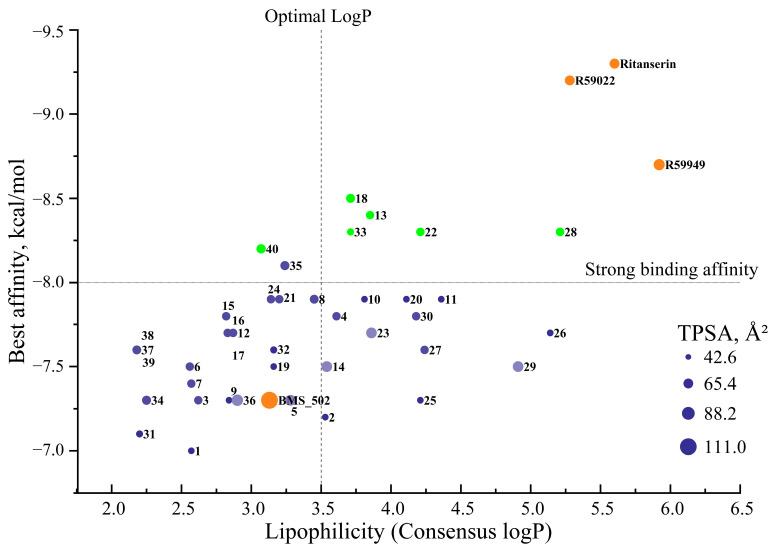
Structure–property relationships of the novel spiro[1,2,4]triazolo[1,5-*c*]quinazoline derivatives and reference DGK inhibitors. The scatter plot illustrates the relationship between binding affinity (kcal/mol) and lipophilicity (consensus logP), with the point size representing the topological polar surface area (TPSA). High-affinity compounds (**13**, **18**, **22**, **28**, **33**, and **40**, highlighted in green) demonstrate balanced physicochemical profiles compared to the reference inhibitors (orange). Blue shades for other compounds are based on the TPSA values. The vertical dashed line indicates the optimal logP range (3.5) for drug-like molecules, while the horizontal line at −8.0 kcal/mol indicates the threshold for strong binding affinity. Typical interpretations consider Vina scores below −8.0 kcal/mol as strong binding, −6.5 to −8.0 kcal/mol as moderate binding, and above −6.5 kcal/mol as weak binding. The commonly used threshold of −6.0 kcal/mol is derived from observations across multiple studies, for instance, refs. [17,18], and relative comparisons within a specific system are generally more informative than absolute values.

The comprehensive analysis of physicochemical properties, drug-likeness parameters, and lipophilicity profiles collectively provides a solid foundation for prioritizing compounds for synthesis and biological evaluation. Based on this integrated assessment, compounds **13**, **18**, and **40** (green dots) emerge as particularly promising candidates among several others (Figure 6), combining strong binding affinity with favorable physicochemical profiles conducive to drug development.

In addition to lipophilicity, aqueous solubility represents a critical parameter for drug development, influencing formulation strategies, bioavailability, and dosing regimens. The solubility profiles of the novel spiro[1,2,4]triazolo[1,5-*c*]quinazoline derivatives were evaluated using three complementary computational models: ESOL, Ali, and SILICOS-IT (Table S4).

The predicted solubility values demonstrated significant variability across the series, with compounds classified from “soluble” to “poorly soluble”, depending on their structural features and the prediction model employed. This heterogeneity in solubility profiles suggests opportunities for structural optimization to enhance aqueous solubility while maintaining target binding.

The ESOL model, which typically provides reliable solubility estimates for drug-like molecules, predicted that compounds **1**–**11**, **31**, **34**, and **37**–**39** would exhibit the “soluble” classification (10^−4^ to 10^−3^ mol/L). These compounds, characterized by nitrogen-containing heterocycles or smaller ring systems, demonstrated more favorable solubility profiles than their more lipophilic counterparts.

The Ali model generally predicted higher solubility values for most compounds, with 19 derivatives classified as “soluble”. This model indicated that pyridine-containing compounds (**37**–**39**) and those with piperidine scaffolds (**31**–**40**) demonstrated particularly favorable solubility characteristics, with predicted concentrations approaching 10^−3^ mol/L.

Conversely, the SILICOS-IT model provided more conservative solubility estimates, with the majority of compounds classified as “moderately soluble” or “poorly soluble”. This model identified compounds **26**, **28**, and **29** as particularly problematic from a solubility perspective, with predicted concentrations below 10^−7^ mol/L, suggesting potential challenges for formulation and bioavailability. All three models consistently identified compounds **26**, **28**, and **29** as having the poorest solubility profiles, which aligns with their high lipophilicity values noted in Table S3 and the drug-likeness violations observed in Table S6.

These compounds, characterized by extended aromatic systems and bulky substituents, may require solubility-enhancing formulation strategies or structural modifications to improve their pharmaceutical properties. Among the compounds identified as most promising based on binding affinity, varying solubility profiles were observed. Compound **40**, despite its strong binding in the 97 Å^3^ cavity, exhibited moderate solubility according to the ESOL and Ali models (10^−5^ mol/L) but poor solubility by the SILICOS-IT prediction (8.95 × 10^−8^ mol/L). Similarly, compound **13** showed moderate solubility by the ESOL and Ali models but poor solubility according to SILICOS-IT (4.49 × 10^−8^ mol/L). Compound **33**, which combines strong binding with the adamantyl substituent, demonstrated moderate solubility across all three models (3.60 × 10^−6^ to 3.85 × 10^−6^ mol/L by ESOL and Ali), suggesting a more balanced physicochemical profile. In contrast, compound 22 exhibited borderline moderate to poor solubility, with values ranging from 1.65 × 10^−6^ mol/L (ESOL) to 2.42 × 10^−8^ mol/L (SILICOS-IT).

The discrepancies observed between different solubility prediction models highlight the importance of experimental verification in subsequent development stages. Nevertheless, the computational predictions provide valuable guidance for prioritizing compounds and identifying potential solubility challenges early in the development process. Based on this integrated assessment, compounds **13**, **18**, **33**, and **40** emerge as particularly promising candidates, balancing strong binding affinity with acceptable physicochemical profiles conducive to drug development, though potential solubility challenges may necessitate appropriate formulation strategies.

#### 2.2.4. Drug Development Considerations

**Figure 7:**
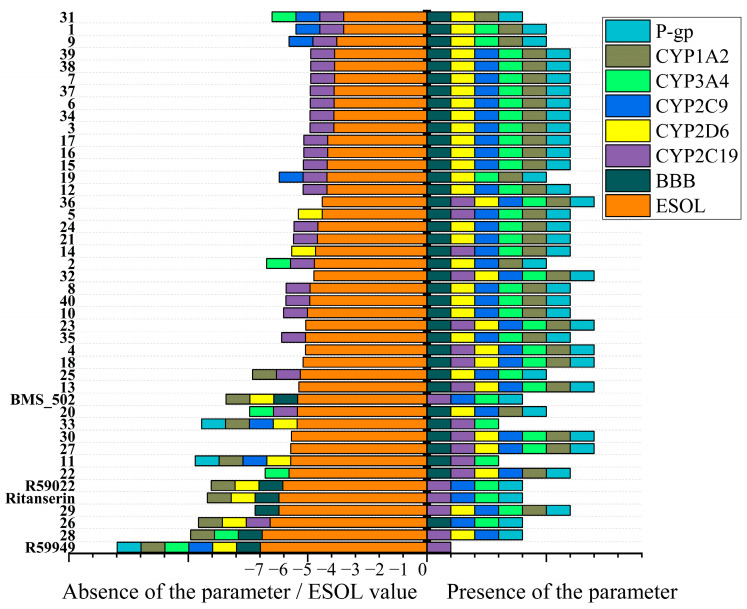
Comparative pharmacokinetic (P-gp substrate status, blood–brain barrier penetration, and CYP inhibition profiles) alongside the ESOL solubility profiles of the novel spiro[1,2,4]triazolo[1,5-*c*]quinazoline derivatives and reference DGK inhibitors (BMS502, R59022, ritanserin, and R59949).

Beyond physicochemical properties and solubility parameters, the prediction of pharmacokinetic behavior and potential drug–drug interactions provides critical insights for prioritizing compounds in the drug development pipeline. Figure 7 (Table S5) presents the predicted ADME (absorption, distribution, metabolism, and excretion) properties and potential cytochrome P450 (CYP) interactions for the novel spiro[1,2,4]triazolo[1,5-*c*]quinazoline derivatives.

All compounds in the series were predicted to exhibit high gastrointestinal absorption, suggesting favorable oral bioavailability potential. This consistent prediction aligns with the moderate molecular weight range (252–412 g/mol) and appropriate lipophilicity profiles observed for most compounds in the series. However, significant variations were observed in other pharmacokinetic parameters, particularly regarding blood–brain barrier (BBB) penetration and CYP inhibition patterns. The majority of compounds (**37** out of **40**) were predicted to penetrate the blood–brain barrier, making them potentially suitable for targeting CNS disorders. Notable exceptions included compounds **28** and **29**, which, despite their high lipophilicity, were predicted to have limited BBB penetration.

This unexpected finding suggests that structural features beyond simple lipophilicity, such as the specific molecular shape or hydrogen bonding patterns, may influence the BBB permeability of these compounds. Most compounds were predicted to be P-glycoprotein (P-gp) substrates, with exceptions including compounds **11** and **33**, which share similar structural features, including bulky hydrophobic substituents. P-gp substrates may experience reduced brain exposure due to active efflux transport at the BBB, potentially limiting their CNS efficacy. However, this property could be advantageous for targeting peripheral tissues while minimizing CNS side effects.

The analysis of potential cytochrome P450 interactions revealed diverse inhibition patterns across the series. Notably, compounds **13**, **18**, **23**, **27**, **30**, and **36** were predicted to inhibit all five major CYP isoforms (1A2, 2C19, 2C9, 2D6, and 3A4), suggesting a high potential for drug–drug interactions. In contrast, compounds **11** and **33** demonstrated more selective CYP inhibition profiles, affecting only CYP2C19 and CYP3A4, which may indicate a reduced potential for drug–drug interactions. Skin permeability (Log Kp) values ranged from −3.85 cm/s (**26**) to −6.60 cm/s (**38** and **39**), with more negative values indicating reduced permeability. Compounds with higher lipophilicity generally exhibited less negative Log Kp values, consistent with enhanced membrane permeation. This parameter is particularly relevant for potential topical applications or assessing systemic exposure following dermal contact.

Among the compounds identified as most promising based on binding affinity, varying pharmacokinetic profiles were observed. Compound **13** exhibited a BBB penetration capability and high gastrointestinal absorption but demonstrated inhibition of all five CYP isoforms, suggesting potential drug–drug interaction concerns. Similarly, compound **18** showed favorable BBB penetration but inhibited multiple CYP enzymes. Compound **40**, which demonstrated strong binding in the 97 Å^3^ cavity, exhibited a more balanced profile with a BBB penetration capability and inhibition of CYP2C9, 2D6, and 3A4, but not 1A2 or 2C19. This more selective CYP inhibition pattern may indicate a reduced potential for drug–drug interactions compared to compounds **13** and **18**.

Perhaps most remarkably, compound **33** (adamantyl-substituted) exhibited a particularly favorable pharmacokinetic profile, with a BBB penetration capability, absence of P-gp substrate properties, and selective inhibition of only CYP2C19 and 3A4. This profile, combined with its strong binding affinity and balanced physicochemical properties, further supports compound **33** as a particularly promising candidate for further development. The comparison with the reference compounds provides additional context for interpreting these predictions.

The integrated analysis of binding affinity, physicochemical properties, and predicted pharmacokinetic parameters provides a foundation for prioritizing compounds for synthesis and biological evaluation. Based on this multidimensional assessment, compound **33** emerges as particularly promising, offering an optimal balance of strong binding affinity, favorable physicochemical properties, and a desirable pharmacokinetic profile with reduced potential for drug–drug interactions. Compounds **13**, **18**, and **40** also represent valuable candidates, though their broader CYP inhibition profiles may necessitate a careful consideration of potential drug–drug interactions.

To complete the multidimensional pharmaceutical evaluation of these novel derivatives, an assessment of bioavailability, synthetic accessibility, and potential structural alerts was conducted (Table S6). The synthetic assessment of availability values, which estimate the relative ease of chemical synthesis, ranged from 3.50 (**1**) to 6.55 (**33**), with lower values indicating more favorable synthetic accessibility. Among the compounds identified as most promising based on binding affinity, varying synthetic complexity was observed. Compounds **13** and **18** demonstrated moderate synthetic complexity (3.88 and 3.77, respectively), suggesting reasonable synthetic feasibility. In contrast, compounds **22** and **40** exhibited slightly higher synthetic complexity (3.99 and 3.98), while compound **33** showed the highest synthetic complexity score (6.55) among all compounds, potentially reflecting challenges associated with incorporating the adamantyl moiety.

Notably, none of the compounds triggered Brenk or PAINS (Pan-Assay Interference Compounds) structural alerts. This finding is particularly significant, as it suggests that the novel derivatives lack substructures associated with false positives in biochemical screening or problematic reactive functionalities, supporting their potential as reliable candidates for biological evaluation. The lead compound assessment revealed that 15 out of the 40 compounds (**1**, **3**, **6**, **7**, **9**, **12**, **15**–**17**, **31**, **34**, and **36**–**39**) met all industry-standard drug-likeness criteria with zero violations. The remaining compounds showed violations primarily related to lipophilicity (XLOGP3 > 3.5) and, in some cases, molecular weight (MW > 350). These violations align with the observations from Table 4, reflecting the inherent lipophilicity of the spiro[1,2,4]triazolo[1,5-*c*]quinazoline scaffold and the substantial molecular weight of the more structurally complex derivatives.

Among the most promising compounds based on binding affinity, compound **40** showed two violations (MW > 350 and XLOGP3 > 3.5), compound **22** exhibited the same two violations, and compound 13 showed a single violation (XLOGP3 > 3.5). Compound **33**, despite its exceptional binding profile and favorable pharmacokinetic properties, demonstrated two violations (MW > 350 and XLOGP3 > 3.5), reflecting its substantial molecular weight (389.54 g/mol) and high lipophilicity (XLOGP3 = 4.72).

The compounds with pyridine substituents (**37**–**39**) displayed the most favorable overall profiles in the lead compound assessment, meeting all drug-likeness criteria with zero violations while maintaining reasonable synthetic complexity (3.74–3.80) and moderate binding affinities. This finding suggests that nitrogen-containing heterocyclic substituents may offer an optimal balance of drug-like properties and synthetic feasibility within this chemical series.

The integrated analysis across Tables S1–S6 provides a comprehensive, multidimensional evaluation of these novel spiro[1,2,4]triazolo[1,5-*c*]quinazoline derivatives. Based on this holistic assessment, compounds **13**, **18**, and **40** emerge as balanced candidates for further development, offering strong binding affinity combined with acceptable pharmaceutical profiles despite minor drug-likeness violations. Compound **33**, while exhibiting higher synthetic complexity, represents a particularly intriguing candidate due to its exceptional binding affinity, favorable pharmacokinetic profile, and the absence of P-gp substrate properties.

### 2.3. Comparative Analysis with the Reference DGK Inhibitors

#### 2.3.1. Comparison with Established DGK Inhibitors

Our comparative analysis focuses on well-established DGK inhibitors (Figure 1) that serve as important benchmarks for evaluating novel derivatives. These reference compounds include ritanserin, a 5-HT2R antagonist with structural similarity to R59022 that also exhibits DGK inhibitory properties [9,23]; R59949, a more potent analog of R59022 [8]; R59022, one of the first reference DGK inhibitors [5,7,9,13]; (5*Z*,2*E*)-CU-3, a DGK-α-selective inhibitor [24]; and BMS502, which targets multiple DGK isoforms, including DGK-α, DGK-ζ, and DGK-ι [12]. Each of these compounds possesses distinctive structural features and inhibitory profiles that inform the design and evaluation of our novel spiro[1,2,4]triazolo[1,5-*c*]quinazoline derivatives.

An examination of these novel derivatives in comparison with the established DGK inhibitors reveals significant distinctions across physicochemical properties, drug-likeness parameters, lipophilicity, solubility, pharmacokinetics, and binding characteristics that collectively inform their potential therapeutic applications.

*Physicochemical property comparison.* Recent phylogenetic analyses have identified multiple conserved sequence signatures that distinguish different DGK classes, demonstrating clear evolutionary divergence in the catalytic domain structure while maintaining strong conservation within classes [25]. This structural divergence is reflected in our comparative analysis of reference compounds versus novel derivatives.

The reference compounds demonstrate substantially larger molecular frameworks (MW: 459.58–516.50 g/mol; heavy atoms: **33**–**38**) compared to our novel derivatives (MW: 252.31–412.53 g/mol; heavy atoms: **19**–**31**). This dimensional differential may confer advantages in membrane permeability and bioavailability to the spiro[1,2,4]triazolo[1,5-*c*]quinazoline series. Notably, the reference inhibitors exhibit predominantly planar structures with limited three-dimensionality (Csp^3^: 0.21–0.26), while several novel compounds, particularly **10**, **11**, **20**, **26**, **32**, and **33**, demonstrate significantly higher Csp^3^ values (≥0.58). This enhanced three-dimensionality has been associated with improved selectivity profiles and reduced promiscuous binding in contemporary drug development paradigms.

The reference compounds’ conformational flexibility (five rotatable bonds versus one–two in novel derivatives) and elevated hydrogen bond acceptor counts (four–eight versus two–four) further distinguish these chemical classes. The topological polar surface area values of the reference compounds (65.85–110.98 Å^2^) generally exceed those of the novel derivatives (42.74–74.22 Å^2^), with BMS502 (110.98 Å^2^) approaching the threshold associated with limited membrane permeability. This observation suggests that our novel compounds may demonstrate superior passive diffusion properties while maintaining adequate polarity for aqueous solubility.

These physicochemical differences align with evolutionary evidence showing distinct sequence signatures in DGK catalytic domains [25], suggesting that our novel derivatives may interact with conserved binding regions in ways that differ from the traditional reference compounds. The molecular profiles of our compounds, particularly their enhanced three-dimensionality and optimized surface areas, may enable more specific targeting of evolutionarily conserved catalytic domain features.

*Drug-likeness.* An examination of drug-likeness criteria reveals substantial differences in compliance profiles between reference and novel compounds. While most novel derivatives (compounds **1**–**10**, **12**–**19**, **21**, **23**, **24**, and **31**–**40**) demonstrated excellent adherence to all five medicinal chemistry filters, the reference compounds exhibited multiple violations. BMS502 failed to meet two Ghose criteria (MW > 480 and MR > 130) and violated Lipinski’s Rule of Five (MW > 500). Similarly, R59022, R59949, and ritanserin each violated multiple filters, particularly relating to lipophilicity and molecular refractivity thresholds.

Most notably, R59949 violated three Ghose criteria (MW > 480, WLOGP > 5.6, and MR > 130), exceeded Egan’s lipophilicity threshold (WLOGP > 5.88), and failed Muegge’s filter (XLOGP3 > 5). This contrasts sharply with the novel derivatives **13**, **18**, and **40**, which demonstrated complete compliance across all drug-likeness filters while maintaining strong binding affinities. The significantly improved drug-likeness profiles of the novel compounds suggest enhanced developability potential compared to the existing DGK inhibitors.

*Lipophilicity profiles.* The lipophilicity comparison revealed distinctive patterns between the reference and novel compounds. The reference inhibitors displayed notably high consensus logP values (BMS502, 3.13; R59022, 5.28; R59949, 5.92; and ritanserin, 5.60) compared to many of the novel derivatives. This elevated lipophilicity in the reference compounds, which was particularly evident in the SILICOS-IT method (R59949, 7.57 and ritanserin, 7.08), suggests potential issues with aqueous solubility and excessive tissue distribution.

In contrast, several high-affinity novel compounds demonstrated more balanced lipophilicity profiles: compound **13** (consensus logP 3.85), compound **18** (consensus logP 3.71), and compound **40** (consensus logP 3.07). These moderate lipophilicity values, approaching the generally accepted optimal range of 2–3.5 for orally administered drugs, suggest more favorable pharmacokinetic behaviors without compromising membrane permeability. The outlier compound **33**, despite its excellent binding profile, exhibited a higher consensus logP (3.71) that remains significantly lower than the reference inhibitors.

*Solubility characteristics.* Solubility predictions by three complementary models (ESOL, Ali, and SILICOS-IT) revealed substantial differences between the reference and novel compounds. The reference compounds consistently demonstrated poor solubility classifications, with particularly problematic profiles for R59949 and ritanserin. R59949 was classified as “poorly soluble” across all three models, with predicted concentrations ranging from 1.05 × 10^−7^ mol/L (ESOL) to 3.09 × 10^−10^ mol/L (SILICOS-IT), suggesting significant formulation challenges.

In contrast, several novel derivatives, particularly those with pyridine substituents (**37**–**39**) and piperidine scaffolds (**31**–**40**), exhibited more favorable solubility characteristics. Compound **33**, despite incorporating the adamantyl moiety, maintained moderate solubility across all prediction models (3.60 × 10^−6^ to 3.85 × 10^−6^ mol/L by ESOL and Ali), suggesting a more balanced physicochemical profile than the reference inhibitors. The improved solubility characteristics of selected novel compounds may confer advantages in formulation flexibility and absorption consistency.

*Pharmacokinetic parameters.* A comparison of the predicted ADME properties revealed significant distinctions in potential pharmacokinetic behavior. While all compounds (reference and novel) were predicted to exhibit high gastrointestinal absorption, notable differences emerged in other parameters. The reference compounds BMS502, R59022, and ritanserin were predicted to penetrate the blood–brain barrier, but R59949 was not, despite its high lipophilicity.

More striking differences appeared in the cytochrome P450 inhibition profiles. The reference compounds demonstrated specific inhibition patterns: BMS502 inhibited CYP1A2, CYP2C19, and CYP3A4; R59022 inhibited CYP1A2, CYP2C19, and CYP3A4; R59949 inhibited CYP1A2; and ritanserin inhibited CYP1A2, CYP2C19, and CYP3A4. In contrast, compound **33** exhibited a more selective inhibition profile (affecting only CYP2C19 and CYP3A4) compared to most other compounds in both the reference and novel series.

Skin permeability coefficients (Log Kp) for the reference compounds (−6.88 to −4.81 cm/s) generally indicated reduced permeability compared to several novel derivatives, particularly those with high lipophilicity. The collective pharmacokinetic analysis suggests that while reference compounds demonstrate established DGK inhibitory activity, selected novel derivatives, particularly compound **33**, may offer improved pharmacokinetic profiles with a reduced drug–drug interaction potential.

*Toxicity classification difference.* The reference compounds generally show LD_50_ values in the range of 900–1600 mg/kg, placing them predominantly in Class IV toxicity. This is comparable to many novel derivatives, though some novel compounds demonstrate potentially improved safety profiles with higher LD_50_ values.

*Organ-specific toxicity profiles*. The reference compounds display distinctive toxicological signatures compared to the novel derivatives:

- R59949 shows higher mutagenicity probability (0.75) compared to most novel compounds (typically <0.61);
- Ritanserin and R59022 both exhibit cytotoxicity probabilities (0.67) that exceed those of the novel derivatives (0.53–0.60);
- BMS502 uniquely demonstrates a higher mutagenicity probability (0.91/yes) compared to all novel compounds, which consistently showed “no” predictions for mutagenicity.

*Balanced toxicity parameters*. While the reference compounds show strong binding affinity, they generally demonstrate less favorable toxicological profiles across multiple endpoints. The novel derivatives, particularly compounds **13**, **18**, **33**, and **40**, maintain comparable binding affinity while exhibiting improved safety profiles with lower probabilities across the hepatotoxicity, carcinogenicity, and mutagenicity endpoints.

*Prediction accuracy considerations.* The toxicity predictions for the reference compounds benefit from their established pharmacological profiles and extensive experimental data, potentially increasing prediction reliability. Nevertheless, the consistent prediction patterns observed for the novel derivatives suggest reliable safety projections that merit experimental verification.

*Binding affinity comparison*. The most significant comparative dimension emerges in the analysis of binding affinities across multiple cavities. While the reference inhibitors demonstrated strong binding in specific cavities (ritanserin, −9.3 kcal/mol in 127 Å^3^; R59022, −9.2 kcal/mol in 127 Å^3^; and R59949, −8.7 kcal/mol in 127 Å^3^, −8.1 kcal/mol in 121 Å^3^), several novel derivatives exhibited comparable or superior binding in alternative cavities.

Compound **18** achieved exceptional binding in the 179 Å^3^ cavity (−8.5 kcal/mol), surpassing all reference compounds in this specific binding region. Similarly, compound **40** demonstrated superior affinity in the 97 Å^3^ cavity (−8.2 kcal/mol), outperforming all reference compounds in this pocket. Compounds **13**, **22**, and **33** (all −8.3 to −8.4 kcal/mol in the 127 Å^3^ cavity) approached the potency of the reference inhibitors while maintaining improved physicochemical profiles.

The enhanced binding of novel derivatives in specific cavities, coupled with their improved drug-like properties, suggest potential advantages in selectivity and reduced off-target effects compared to the reference inhibitors. The differential binding patterns across cavities further indicate opportunities for developing cavity-specific inhibitors with optimized pharmacological profiles.

*Synthetic accessibility.* An assessment of synthetic feasibility revealed substantial differences between the reference and novel compounds. The reference inhibitors demonstrated moderate synthetic complexity (BMS502, 3.81; R59022, 3.73; R59949, 3.67; and ritanserin, 3.73), comparable to many novel derivatives. However, significant variations existed within the novel series, with compounds **1** and **7** offering the most favorable synthetic routes (scores of 3.50) and compound **33** presenting greater synthetic challenges (score of 6.55).

Importantly, several compounds with excellent binding profiles (**13**—3.88; **18**—3.77; and **40**—3.98) demonstrated a synthetic complexity comparable to the reference inhibitors, suggesting feasible synthetic approaches for these promising candidates. This analysis provides valuable guidance for prioritizing compounds based on the balance between binding potency and synthetic accessibility.

*Integrated comparative assessment.* The multidimensional comparative analysis reveals that while reference DGK inhibitors (BMS502, R59022, R59949, and ritanserin) demonstrate established activity, they exhibit several pharmaceutical limitations, including an excessive molecular weight, high lipophilicity, poor predicted solubility, and multiple drug-likeness violations. In contrast, selected novel spiro[1,2,4]triazolo[1,5-*c*]quinazoline derivatives, particularly compounds **13**, **18**, **33**, and **40**, offer a more balanced profile combining strong binding affinity with improved physicochemical and pharmacokinetic properties.

Compound **33** emerges as especially noteworthy, demonstrating exceptional binding affinity (−8.3 kcal/mol in the 127 Å^3^ cavity), a favorable pharmacokinetic profile (selective CYP inhibition and the absence of P-gp substrate properties), moderate solubility, and enhanced three-dimensionality (Csp^3^: 0.67), despite its synthetic complexity. Similarly, compound **13** offers an optimal balance of strong binding (−8.4 kcal/mol in the 127 Å^3^ cavity), favorable drug-likeness, acceptable lipophilicity (consensus logP 3.85), and reasonable synthetic accessibility (score of 3.88).

It worth noting that ritanserin is used in medicine [9,23], while having violations of drug-likeness rules. This observation highlights an important consideration in modern pharmaceutical development regarding the evolution of drug discovery paradigms and the retrospective application of current screening criteria to established medications. It represents a compound developed prior to the widespread implementation of the rigorous drug-likeness filters that characterize contemporary pharmaceutical research. The violations observed reflect physicochemical properties that would indeed raise concerns in current drug discovery programs, particularly regarding oral bioavailability and metabolic stability. However, several factors contribute to ritanserin’s continued clinical relevance, despite these apparent limitations.

Current research indicates that drug-likeness rules, while being valuable screening tools, represent statistical guidelines rather than absolute requirements for therapeutic efficacy. Preliminary evidence suggests that compounds may achieve clinical success through alternative mechanisms, including specialized delivery methods, targeted therapeutic applications, or unique pharmacokinetic profiles, that compensate for apparent violations. Furthermore, ritanserin’s established clinical use demonstrates that certain violations may be tolerable when balanced against the therapeutic benefit and when appropriate formulation strategies are employed.

The significance of our novel derivatives lies in their ability to maintain comparable binding affinity while eliminating these physicochemical violations, thereby combining therapeutic potential with improved drug-like properties. Compounds **13**, **18**, **33**, and **40** demonstrate significant improvements over ritanserin by achieving more balanced physicochemical profiles that should theoretically result in enhanced pharmacokinetic behaviors, a reduced metabolic burden, and improved safety margins. This represents a meaningful advancement in the optimization of DGK inhibitor design. Further investigation through experimental validation is warranted to confirm that our optimized compounds demonstrate superior pharmacological profiles compared to existing therapeutic agents.

#### 2.3.2. Comparison with Representatives of Patent CN 115362003 B

Patent CN 115362003 B [10] discloses a broad array of [1,2,4]triazolo[4,3-*a*]quinazolin-5-amine derivatives (Figure 2) as potential DGK-α modulators. However, several factors influenced our decision to focus primarily on the novel spiro-fused derivatives rather than comprehensively evaluating them toward all patent compounds: (1) The patent encompasses numerous structural variations that would have substantially expanded the computational requirements beyond the scope of this investigation. (2) These compounds are claimed to be designed to modulate DGK-α, but without experimental validation of biological data or detailed structure-activity relationships, that would enable a meaningful correlation between molecular structure and binding affinity. They have wide potency range—spanning from sub-nanomolar (0.38 nM) to values exceeding 10,000 nM within the same compound series, which represents an unusually extensive activity spectrum rarely encountered in kinase inhibitor development. (3) The primary research objective was to develop a distinct structural class with improved pharmacological profiles through significant scaffold modification rather than iterative optimization of existing chemotypes. (4) The spiro modification represents a fundamental structural transformation that creates a unique three-dimensional architecture specifically designed to enhance molecular complexity for improved selectivity and pharmaceutical properties.

Nevertheless, it was decided to conduct molecular docking studies with two representative patent compounds—*N*-methyl-[1,2,4]triazolo[4,3-*a*]quinazolin-5-amine and compound **p523** (8-chloro-5-(5-(cyclopropylethynyl)-3,4-dihydroquinolin-1(2*H*)-yl)-7-fluoro-[1,2,4]triazolo[4,3-*a*]quinazoline)—to establish critical reference points for evaluating the impacts of the presented structural modifications. The simple *N*-methyl derivative represents the basic patent scaffold, while compound **p523** was specifically selected for its lowest reported IC_50_ of 1 nM and EC_50_ of 0.24 nM [10].

The main patent scaffold demonstrates modest binding affinity across all five cavities (Table 5), with Vina scores ranging from −4.6 to −6.2 kcal/mol. Compound **p523**, despite being reported as highly potent in the patent (IC_50_ of 1 nM), shows improved, but still moderate computational binding scores (−6.6 to −7.5 kcal/mol) that fail to fully correlate with its exceptional reported biological activity.

In contrast, the spiro-modified derivatives consistently exhibit superior binding profiles across all cavities. This enhanced binding can be attributed to several key structural advantages:

- The spiro fusion creates a unique three-dimensional architecture that optimizes the spatial orientation within the binding pockets, which is particularly evident in the 179 Å^3^ cavity, where compound **18** achieves a remarkable −8.5 kcal/mol binding score.
- The introduction of conformational constraint through spiro-ring systems appears to position key pharmacophore elements for optimal interaction with critical protein residues, as demonstrated by compound **13**’s excellent binding (−8.2 kcal/mol) in the 179 Å^3^ cavity.
- The incorporation of heterocyclic substituents in the spiro-derivatives provides additional hydrogen bonding capabilities and π–electron interactions that are absent in the basic scaffold, contributing to compound **33**’s enhanced binding (−8.2 kcal/mol) in the 179 Å^3^ cavity.
- The adamantyl-substituted compound **33** demonstrates particularly strong binding across multiple cavities, suggesting that its unique spatial geometry and lipophilic character create favorable interactions within the hydrophobic regions of the binding sites.
- Compound **40** shows exceptional versatility with strong binding across all cavities, including an optimal interaction (−8.2 kcal/mol) in the 127 Å^3^ cavity, indicating its balanced pharmacophore arrangement.

Furthermore, while the patent compounds like **p453** incorporate halogens and ethynyl substituents that may compromise pharmaceutical properties, the presented spiro-modified approach achieves superior overall binding profiles while maintaining favorable drug-like characteristics, as evidenced by the comprehensive physicochemical analysis presented in Section 2.2.

This integrated comparison suggests that the novel spiro[1,2,4]triazolo[1,5-*c*]quinazoline derivatives represent a substantial advancement over the existing DGK inhibitors, offering improved pharmaceutical profiles while maintaining comparable or superior binding capabilities. These findings provide a robust foundation for the further development of these compounds as next-generation DGK modulators with enhanced therapeutic potential.

### 2.4. Structure–Activity Relationships

**Figure 8:**
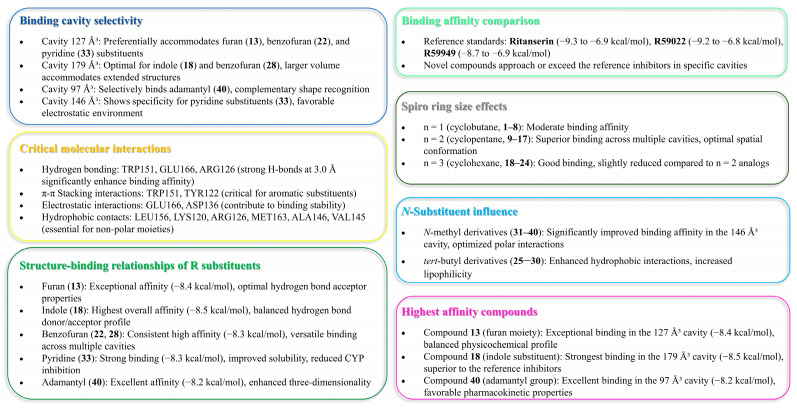
Structure–activity relationships of the spiro[1,2,4]triazolo[1,5-*c*]quinazoline derivatives as DGK-α modulators: key structural features and binding determinants.

The systematic computational analysis revealed critical structural determinants that significantly influence binding affinity, physicochemical properties, and pharmacokinetic parameters. Figure 8 illustrates the identified structure–activity relationships (SARs).

The [1,2,4]triazolo[1,5-*c*]quinazoline core scaffold provides the essential framework for DGK-α binding, while three key regions allow for strategic modifications: the spiro ring size, the scaffold type (piperidine or cyclohexane), and the heterocyclic substituent. Our analysis demonstrates that each of these regions contributes distinctively to the overall molecular properties and target engagement.

The identification of these structure–activity relationships is supported by evolutionary evidence showing that DGK catalytic domains maintain distinct sequence signatures within classes while showing clear divergence between classes [25]. This evolutionary conservation pattern aligns with observations of class-specific binding preferences and interaction patterns, particularly for the DGK-ε, -θ and -ζ isoforms, which show a broad distribution across eukaryotic lineages. The conservation of specific binding pocket architectures across evolutionary time suggests that the targeting approach exploits fundamental features of DGK catalytic domains.

The spiro ring size emerged as a critical determinant of binding affinity. Derivatives containing an n = 2 system (cyclopentane, compounds **9**–**17**) consistently exhibited superior binding compared to their n = 1 (cyclobutane) or n = 3 (cyclohexane) counterparts. This optimal ring size likely provides the ideal spatial orientation for engaging key binding residues within the DGK-α protein structure, particularly in the 127 Å^3^ and 179 Å^3^ cavities that showed the strongest binding potential.

The heterocyclic substituent significantly influenced both the binding affinity and physicochemical properties. Oxygen-containing heterocycles demonstrated exceptional binding characteristics, with the furan-containing compound **13** exhibiting the strongest binding among novel derivatives in the 127 Å^3^ cavity (−8.4 kcal/mol) and benzofuran derivatives (**22** and **28**) showing excellent binding across multiple cavities. Nitrogen-containing heterocycles provided distinctive binding profiles, with the indole-substituted compound **18** achieving the highest binding affinity in the 179 Å^3^ cavity (−8.5 kcal/mol) and pyridine-containing derivatives (**30** and **33**) showing favorable binding coupled with improved physicochemical properties. The adamantyl group (**33**) produced one of the most promising candidates, combining strong binding affinity with selective CYP inhibition profiles.

Modification of the piperidine/cyclohexane scaffold revealed that the presence of a *tert*-butyl group enhanced binding through additional hydrophobic interactions, while piperidine-containing compounds (**31**–**40**) demonstrated higher hydrogen bond acceptor capacities that influenced both binding and solubility profiles.

A detailed molecular interaction analysis identified key residues critical for high-affinity binding. Compounds forming stronger hydrogen bonds with TRP151, GLU166, and ARG126 generally exhibited superior binding profiles. Notably, compound **40** achieved its exceptional binding in the 97 Å^3^ cavity through a much stronger conventional hydrogen bond with GLU166 (1.85 Å) compared to the reference inhibitors, highlighting the importance of optimal hydrogen bonding networks.

The interplay between structural modifications and physicochemical properties revealed important trends. Pyridine-substituted derivatives demonstrated improved solubility and lower lipophilicity (consensus logP = 2.18), while benzofuran-substituted compounds showed higher binding affinity but increased lipophilicity (logP > 5). Compounds with higher fractions of sp^3^ hybridized carbon atoms (Csp^3^ ≥ 0.58), particularly compounds **10**, **11**, **20**, **26**, **32**, and **33**, exhibited enhanced three-dimensionality, which has been associated with improved selectivity profiles.

The pharmacokinetic analysis revealed that most compounds were predicted to penetrate the blood–brain barrier, with the notable exception of compounds **28** and **29**, despite their high lipophilicity. Compound **33** emerged as particularly promising, demonstrating selective CYP inhibition (affecting only CYP2C19 and CYP3A4) and the absence of P-gp substrate properties, suggesting a reduced potential for drug–drug interactions.

Based on this SAR analysis, compounds **13**, **18**, **33**, and **40** represent the most promising candidates for further development, offering a balanced profile of strong binding affinity and favorable physicochemical and pharmacokinetic properties. These findings provide a solid foundation for the rational design of next-generation DGK-α modulators with optimized therapeutic potential.

### 2.5. Limitations

Our computational approach to evaluating novel spiro[1,2,4]triazolo[1,5-*c*]quinazoline derivatives as DGK-α modulators has several key limitations:

1. Methodological

- Absence of experimental validation. The most significant limitation is the lack of experimental validation through biochemical assays. An experimental determination of IC_50_ values, binding constants, and enzyme inhibition profiles would be essential to confirm the predicted activities of these compounds.
- Comprehensive structural modeling. The integration of the complete protein structure may reveal additional interaction sites, particularly within the catalytic domain, where inhibitors like R59949 demonstrate binding affinity.
- Implementation of dynamic models. The application of molecular dynamic simulations could potentially capture protein flexibility and conformational changes during ligand binding, offering more nuanced insights than static docking approaches.

2. *Mechanistic Validation Possibilities*

- Biochemical assay development. To address the validation gap, the development of biochemical assays could provide critical validation parameters (IC_50_, Ki values, and binding kinetics) that may confirm or refine computational predictions.
- Cellular activity assessment. The absence of cell-based evaluations limits our understanding of the compounds’ abilities to penetrate cellular membranes and modulate DGK-α activity in physiologically relevant environments.
- Isoform selectivity assessment. Comprehensive profiling across DGK isoforms might identify compounds with optimal selectivity profiles, potentially minimizing off-target effects that commonly limit kinase inhibitor utility.
- Structure–activity relationship development. Systematic structural modifications could potentially establish clear correlations between molecular features and both binding affinity and selectivity, guiding rational design iterations.

3. *Translational research opportunities*

- Expanded pharmacokinetic evaluation. A detailed investigation of physiological stability, metabolic pathways, and bioavailability profiles could identify candidates with favorable drug-like properties.
- Enhanced CNS penetration analysis. Advanced modeling and experimental validation of blood–brain barrier permeability might identify compounds suitable for neurological applications, particularly through refined QSPR analyses.
- Comprehensive interaction profiling. A broader assessment of potential drug–drug interactions, including transporter effects and pharmacodynamic consequences, could identify compounds with favorable clinical profiles.

4. *Technical advancement possibilities*

- Scoring function diversification. The application of multiple complementary evaluation methods might yield more robust binding predictions by mitigating algorithm-specific biases.
- Advanced simulation implementation. Extended molecular dynamics with enhanced sampling techniques could potentially provide deeper insights into the stability and kinetics of predicted protein–ligand complexes.
- Quantum mechanical modeling integration. The incorporation of QM/MM approaches might more accurately represent electronic effects on critical binding interactions, particularly for compounds with complex electronic distributions.

### 2.6. Future Directions

*Experimental validation of computational predictions*. To address the computational nature of the presented findings, several experimental validation approaches could be considered:

- Direct binding assessment possibilities. Radioligand binding assays using [^3^H]phorbol 12-myristate 13-acetate displacement might provide valuable data on direct interactions with the DGK-α catalytic domain for promising compounds such as **13**, **18**, **33**, and **40**. Surface plasmon resonance experiments could potentially measure binding kinetics and affinity constants, which would offer quantitative validation of predicted binding affinities.
- Enzyme inhibition evaluation options. In vitro DGK-α inhibition assays using purified recombinant human DGK-α could determine IC_50_ values and the inhibitory mechanisms. Thermal shift assays might confirm physical interactions with the target protein and provide insights into binding-induced conformational changes.
- Selectivity investigation avenues. Compounds showing promising activity could be screened against various DGK isoforms to assess their selectivity profiles. Additionally, counter-screening against related kinases might evaluate potential off-target activities.
- Comparative analysis considerations. Competitive binding studies with established inhibitors like R59949 could help determine binding site overlap and provide context for the novel compounds’ inhibitory mechanisms, complementing the computational comparison presented here.

*Dose–response relationship investigation*. A critical next step in developing these DGK-α modulators will be to conduct dose–response studies to establish optimal therapeutic windows. This is particularly important given that DGK inhibition may exhibit bell-shaped dose–response curves, where moderate inhibition may enhance therapeutic effects while complete inhibition could lead to adverse outcomes. We propose investigating concentration ranges from 1 nM to 10 µM across multiple cell lines to determine the IC_50_ values and establish structure–activity relationships in cellular contexts [7,8].

*Additional validation models.* Our findings require validation across multiple experimental models. We propose the following:

- Cell-based assays using both cancer cell lines (particularly glioblastoma, where DGK-α plays a critical role) and immune cells (T cells) to assess effects on cell proliferation and activation [1,4];
- Ex vivo organoid models to evaluate the compounds’ efficacy in more complex tissue environments;
- Animal models of cancer and inflammatory disorders to validate in vivo efficacy [9].

*Structural optimization roadmap.* We propose a logical progression for compound optimization as follows:

- For compounds showing the highest binding affinity (e.g., **18** and **40**), introduce modifications to improve solubility while maintaining target engagement;
- For compounds with balanced pharmacokinetic profiles (e.g., **33**), explore bioisosteric replacements of the adamantyl group to maintain binding while reducing synthetic complexity;
- Develop hybrid structures incorporating the indole moiety from compound **18** with the spiro-piperidine scaffold from compound **33** to potentially combine superior binding with improved pharmacokinetics;
- Introduce polar substituents at specific positions on the quinazoline core to enhance solubility without disrupting key binding interactions [26,27].

*Broader therapeutic applications.* Beyond cancer immunotherapy and the treatment of viral infections mentioned in the original patent (CN 115362003 B) [10], our novel DGK-α modulators may have significant therapeutic potential in the following areas:

- Neurodegenerative disorders [2], where abnormal lipid signaling contributes to disease progression;
- Autoimmune disorders [4], where the modulation of T-cell responses could restore immune homeostasis;
- Cardiac hypertrophy and heart failure [3], where DGK-α plays a role in pathological remodeling;
- Metabolic disorders, particularly type 2 diabetes [2], where altered diacylglycerol signaling affects the insulin response.

Additionally, the novel spiro[1,2,4]triazolo[1,5-*c*]quinazoline derivatives have already demonstrated significant potential for addressing complex neurological and neuropsychiatric conditions through multifaceted molecular mechanisms [28]. The molecular profile suggests potential therapeutic utility in anxiety disorder management, cognitive dysfunction rehabilitation, potential antidepressant interventions, and mitigation of stress-induced neurological impairments.

These expanded applications would significantly broaden the impact of our structural approach to developing DGK-α modulators and provide additional avenues for therapeutic development.

## 3. Materials and Methods

### 3.1. Molecular Docking Studies

#### Computational Framework of the Structure-Based Blind Docking Analysis

The computational approach was informed by the structural complexity of DGK isoforms, recognizing that each subfamily possesses unique regulatory domains that may influence binding interactions. Following Topham and Epand’s [2] framework of DGK structural classification, potential domain-specific interactions were carefully considered during the docking simulations.

We employed the CB-Dock2 web server platform [14,15] to conduct structure-based blind docking analyses. This computational approach integrates two complementary strategies: a curvature-based algorithm that detects potential binding cavities on the protein surface when no suitable templates are available, with subsequent molecular docking via AutoDock Vina (v1.2.0); and a template-based approach that leverages homologous structural information when compounds with significant topological similarity (FP2 similarity score ≥0.4) are identified in the BioLip structural repository (2021.09.15 release). The template-based approach incorporates the hierarchical FitDock algorithm to optimize ligand placement and generate refined binding poses. This dual-pathway methodology enhances the binding site prediction accuracy by integrating evolutionary information when available while maintaining robust performance for novel protein–ligand interactions.

The macromolecule from the RCSB Protein Data Bank (PDB), human DGK-α (PDB ID: 6IIE) in pdb format, served as the biological target for this investigation [16]. Molecular structures of 40 spiro[1,2,4]triazolo[1,5-*c*]quinazoline derivatives (Figure 2) and 5 reference compounds (Figure 1) were constructed using ChemDraw Professional 15.0, saved in mol format, and subsequently subjected to blind docking with DGK-α via the CB-Dock2 web platform [15]. The reference compounds included: **ritanserin** (6-[2-[4-[*bis*(4-fluorophenyl)methylene]-1-piperidinyl]ethyl]-7-methyl-5*H*-thiazolo[3,2-*a*]pyrimidin-5-one), initially developed as a serotonin receptor antagonist [9,23]; **R59022** (6-[2-[4-[(4-fluorophenyl)phenylmethylene]-1-piperidinyl]ethyl]-7-methyl-5*H*-thiazolo[3,2-*a*]pyrimidin-5-one), recognized as one of the first DGK inhibitors [5,7,9,13]; **R59949** (3-[2-[4-[*bis*(4-fluorophenyl)methylene]-1-piperidinyl]ethyl]-2,3-dihydro-2-thioxo-4(1*H*)-quinazolinone), a more potent R59022 analog [8]; **(5Z,2E)-CU-3** (*N*-[(5*Z*)-5-[(2*E*)-3-(2-furanyl)-2-propen-1-ylidene]-4-oxo-2-thioxo-3-thiazolidinyl]-benzenesulfonamide), a DGK-α-specific inhibitor [24]; and **BMS502** (8-[4-[*bis*(4-fluorophenyl)methyl]-1-piperazinyl]-5,6-dihydro-5-methyl-7-nitro-6-oxo-1,5-naphthyridine-2-carbonitrile), which targets the DGK-α, DGK-ζ, and DGK-ι isoforms [12]. The patented *N*-methyl-[1,2,4]triazolo[4,3-*a*]quinazolin-5-amine with **p523** (8-chloro-5-(5-(cyclopropylethynyl)-3,4-dihydroquinolin-1(2*H*)-yl)-7-fluoro-[1,2,4]triazolo[4,3-*a*]-quinazoline) were also included [10].

The computational analysis identified five distinct binding cavities with volumes ranging from 97 Å^3^ to 179 Å^3^, each exhibiting unique geometric properties and interaction potentials (Table 1 and Table 5). The results were downloaded as txt files for each compound. BIOVIA Discovery Studio 2017 R2 facilitated the visualization and analysis of the protein–ligand complexes in pdb format, demonstrating highest binding affinities: ritanserin versus compound **3** in the 127 Å^3^ cavity; R59949 versus compound **18** in the 179 Å^3^ cavity; R59949 versus compound **40** in the 97 Å^3^ cavity; R59949 versus compound **28** in the 121 Å^3^ cavity; and R59022 versus compound **33** in the 146 Å^3^ cavity (Figure 4 and Figure 5, Table 3).

### 3.2. Physicochemical and ADME Property Predictions

The physicochemical property analysis was conducted using SwissADME [19,20], an integrated web tool for medicinal chemistry and drug discovery applications. The molecular structures of all studied and reference compounds were submitted in SMILES format to calculate key physicochemical parameters, including the molecular weight, heavy atom count, aromatic heavy atom count, fraction of sp^3^ hybridized carbon atoms, rotatable bonds, hydrogen bond acceptors and donors, molecular refractivity, and topological polar surface area.

SwissADME was also employed to assess drug-likeness according to multiple industry-standard filters (Lipinski, Ghose, Veber, Egan, and Muegge) and to calculate lipophilicity parameters using five distinct computational methods: iLOGP (internal VCCLAB method), XLOGP3 (atom-based method), WLOGP (atomistic method based on fragmental data), MLOGP (topological method), and SILICOS-IT (hybrid method). Consensus lipophilicity values were calculated as the arithmetic means of these five predictions.

Solubility was evaluated using three complementary computational models, ESOL, Ali, and SILICOS-IT, with the results reported in both mg/mL and mol/L concentrations. Compounds were classified as highly soluble, soluble, moderately soluble, or poorly soluble based on established the threshold values for each model.

The pharmacokinetic property prediction was performed using both SwissADME and ProTox-2 and 3 [21,22] web servers. SwissADME was used to predict gastrointestinal absorption, blood–brain barrier permeation, the P-glycoprotein substrate status, and skin permeability coefficient (Log Kp). ProTox provided complementary toxicity predictions and CYP inhibition profiles for five major cytochrome P450 isoforms: CYP1A2, CYP2C19, CYP2C9, CYP2D6, and CYP3A4. Synthetic accessibility was assessed using SwissADME’s built-in synthetic assessment algorithm, which estimates the relative ease of chemical synthesis on a scale from 1 (very easy to synthesize) to 10 (very difficult to synthesize).

Structural alerts were evaluated using both SwissADME (for Brenk and PAINS alerts) and ProTox for additional toxicophoric identification. Bioavailability was estimated using Abbott’s bioavailability score as implemented in SwissADME, with values closer to 1 indicating a higher probability of >10% oral bioavailability in humans.

All computational predictions were performed with the default parameters as implemented in the respective web servers. The combined use of multiple prediction methodologies for key parameters such as lipophilicity and solubility provided a more robust assessment of the physicochemical and pharmacokinetic properties, reducing the potential bias associated with individual computational models.

## 4. Conclusions

This computational investigation has identified novel spiro[1,2,4]triazolo[1,5-*c*]quinazoline derivatives as promising DGK-α modulators, with compounds **13**, **18**, **33**, and **40** demonstrating an optimal balance of strong target engagement and favorable pharmaceutical profiles. The established structure–activity relationships reveal that oxygen- and nitrogen-containing heterocycles, an optimal spiro ring size (n = 2), and strategic hydrogen bonding with key residues (TRP151, GLU166, and ARG126) significantly enhance binding affinity. These compounds represent valuable starting points for developing next-generation therapeutics targeting DGK-α-mediated signaling pathways in cancer, inflammatory disorders, and viral infections, with potential advantages over existing inhibitors, including improved selectivity and reduced off-target effects.

## Figures and Tables

**Table 1:** Comparative analysis of binding cavity properties and affinity scores (kcal/mol) of the reference DGK-α inhibitors across five distinct binding sites in the DGK-α structure (RCSB PDB ID: 6IIE).

Volume, Å^3^	Center (x, y, z)	Docking Size (x, y, z)	Ritanserin	R59022	R59949	BMS502	(5*Z*,2*E*)-CU-3
**127**	−27.971, 12.861, −7.859	21, 21, 21	−9.3	−9.2	−8.7	−7.3	−6.3
**179**	−16.001, 4.140, 1.890	21, 21, 21	−7.1	−7.3	−7.7	−6.4	−6.5
**97**	−12.904, 16.082, −0.064	21, 21, 21	−7.5	−7.3	−7.6	−6.5	−6.4
**121**	−29.580, 15.955, 7.175	21, 21, 21	−7.8	−6.8	−8.1	−6.2	−5.7
**146**	−21.667, 10.869, 16.559	21, 21, 21	−6.9	−7.1	−6.9	−6.2	−6.2

Note: The background is used for better perception of information. Cavity volumes are reported in cubic Angstroms (Å^3^) and represent the accessible volume of each binding pocket as calculated by the CB-Dock2 algorithm. Center (x, y, z) coordinates define the geometric center of each cavity in the protein structure coordinate system (RCSB PDB ID: 6IIE). Docking size (x, y, z) parameters represent the dimensions of the search box used for molecular docking in each direction, optimized to encompass the entire binding pocket. Vina scores are reported in kcal/mol, with more negative values indicating stronger predicted binding affinity. Cavities are ranked in order of the sum of binding affinities across all reference compounds, with the 127 Å^3^ cavity showing the strongest overall binding potential, suggesting that it may represent the primary inhibitor binding site. The reference compounds represent structurally diverse DGK inhibitors, allowing an assessment of binding pocket preferences across different chemical scaffolds.

**Table 2:** Binding affinity analysis (Vina scores) of novel [1,2,4]triazolo[1,5-*c*]quinazoline derivatives and reference DGK-α inhibitors across five distinct binding cavities, ranked by binding strength.

Cavity Volumes									
**127 Å^3^**		**179 Å^3^**		**97 Å^3^**		**121 Å^3^**		**146 Å^3^**	
**Sub.**	**kcal/mol**	**Sub.**	**kcal/mol**	**Sub.**	**kcal/mol**	**Sub.**	**kcal/mol**	**Sub.**	**kcal/mol**
**Ritans.**	−9.3	**18**	−8.5	**40**	−8.2	**R59949**	−8.1	**33**	−7.5
**R59022**	−9.2	**28**	−8.3	**22**	−8.1	**Ritans.**	−7.8	**11**	−7.3
**R59949**	−8.7	**13**	−8.2	**8**	−7.9	**28**	−7.2	**28**	−7.3
**13**	−8.4	**33**	−8.2	**18**	−7.9	**33**	−7.2	**22**	−7.1
**22**	−8.3	**35**	−8.1	**28**	−7.8	**40**	−7.1	**R59022**	−7.1
**33**	−8.3	**10**	−7.9	**35**	−7.8	**11**	−7.0	**18**	−6.9
**35**	−8.1	**11**	−7.9	**30**	−7.7	**13**	−6.9	**Ritans.**	−6.9
**28**	−8.0	**22**	−7.9	**R59949**	−7.6	**35**	−6.8	**R59949**	−6.9
**11**	−7.9	**40**	−7.9	**4**	−7.5	**R59022**	−6.8	**4**	−6.8
**18**	−7.9	**4**	−7.8	**13**	−7.5	**30**	−6.7	**13**	−6.8
**20**	−7.9	**8**	−7.8	**Ritans.**	−7.5	**18**	−6.6	**35**	−6.8
**21**	−7.9	**15**	−7.8	**29**	−7.4	**22**	−6.6	**40**	−6.8
**24**	−7.9	**30**	−7.8	**24**	−7.3	**26**	−6.6	**8**	−6.7
**10**	−7.8	**12**	−7.7	**27**	−7.3	**4**	−6.4	**24**	−6.6
**40**	−7.8	**16**	−7.7	**R59022**	−7.3	**8**	−6.4	**26**	−6.6
**23**	−7.7	**17**	−7.7	**33**	−7.2	**10**	−6.3	**30**	−6.6
**26**	−7.7	**R59949**	−7.7	**11**	−7.1	**29**	−6.3	**27**	−6.5
**4**	−7.6	**20**	−7.6	**15**	−7.1	**24**	−6.2	**29**	−6.5
**8**	−7.6	**26**	−7.6	**21**	−7.1	**32**	−6.2	**32**	−6.5
**32**	−7.6	**27**	−7.6	**25**	−7.0	**BMS502**	−6.2	**15**	−6.3
**37**	−7.6	**37**	−7.6	**37**	−7	**2**	−6.1	**20**	−6.3
**6**	−7.5	**38**	−7.6	**12**	−6.9	**16**	−6	**23**	−6.3
**19**	−7.5	**39**	−7.6	**26**	−6.9	**27**	−6	**16**	−6.2
**7**	−7.4	**14**	−7.5	**31**	−6.9	**38**	−6	**17**	−6.2
**15**	−7.4	**29**	−7.5	**34**	−6.9	**15**	−5.9	**21**	−6.2
**30**	−7.4	**6**	−7.4	**16**	−6.8	**17**	−5.9	**BMS502**	−6.2
**27**	−7.3	**7**	−7.4	**3**	−6.6	**20**	−5.9	**CU-3**	−6.2
**29**	−7.3	**19**	−7.4	**6**	−6.6	**37**	−5.9	**6**	−6.1
**BMS502**	−7.3	**24**	−7.4	**7**	−6.6	**39**	−5.9	**10**	−6.1
**16**	−7.2	**32**	−7.4	**9**	−6.6	**7**	−5.8	**12**	−6.1
**17**	−7.2	**3**	−7.3	**14**	−6.6	**34**	−5.8	**37**	−6.1
**3**	−7.1	**5**	−7.3	**17**	−6.6	**6**	−5.7	**38**	−6.1
**5**	−7.1	**9**	−7.3	**23**	−6.6	**21**	−5.7	**39**	−6.1
**12**	−7.1	**21**	−7.3	**38**	−6.6	**25**	−5.7	**14**	−6
**25**	−7.1	**25**	−7.3	**10**	−6.5	**36**	−5.7	**25**	−5.9
**38**	−7.1	**34**	−7.3	**36**	−6.5	**CU-3**	−5.7	**31**	−5.9
**1**	−7.0	**36**	−7.3	**39**	−6.5	**9**	−5.6	**34**	−5.9
**2**	−7.0	**R59022**	−7.3	**BMS502**	−6.5	**12**	−5.6	**2**	−5.8
**14**	−7	**2**	−7.2	**1**	−6.4	**14**	−5.6	**7**	−5.8
**39**	−7	**31**	−7.1	**20**	−6.4	**19**	−5.6	**19**	−5.8
**34**	−6.9	**Ritans.**	−7.1	**CU-3**	−6.4	**23**	−5.6	**36**	−5.8
**9**	−6.8	**1**	−7.0	**5**	−6.3	**31**	−5.5	**1**	−5.7
**36**	−6.7	**23**	−7.0	**19**	−6.3	**5**	−5.4	**3**	−5.7
**31**	−6.6	**CU-3**	−6.5	**32**	−6.2	**1**	−5.2	**5**	−5.7
**CU-3**	−6.3	**BMS502**	−6.4	**2**	−6	**3**	−5.2	**9**	−5.6

Note: The background is used for better perception of information. Compounds are presented in descending order of binding affinity (Vina score) within each cavity, allowing a direct comparison of the relative binding potency among all tested compounds. The reference compounds are ritanserin, R59022, R59949, BMS502, and (5*Z*,2*E*)-CU-3. Vina scores are reported in kcal/mol, with more negative values indicating a stronger predicted binding affinity. Scores below −8.0 kcal/mol represent compounds with a particularly strong binding potential. The highest scoring compounds in each cavity (e.g., ritanserin in 127 Å^3^, **18** in 179 Å^3^, **40** in 97 Å^3^, R59949 in 121 Å^3^, and **33** in 146 Å^3^) represent lead candidates for targeting these specific binding sites. Consistently high-scoring compounds across multiple cavities (e.g., **13**, **22**, **28**, **33**, and **35**) suggest molecular features that enable adaptable binding to different protein environments. Reference inhibitors (particularly ritanserin and R59022) establish performance benchmarks, with novel compounds scoring within 1.0 kcal/mol of these references considered to have comparable predicted binding potency. The 127 Å^3^ cavity shows the highest overall binding scores, particularly for the reference compounds, suggesting that it may represent the primary inhibitory binding site for DGK modulators. Some compounds (e.g., **18** and **40**) outperform the reference inhibitors in specific cavities, indicating their potential as selective DGK modulators targeting these distinct binding regions.

**Table 3:** Detailed molecular interaction analysis of the top-performing reference compounds and novel [1,2,4]triazolo[1,5-*c*]quinazoline derivatives in each DGK-α binding cavity, characterizing the interaction types, distances, and specific amino acid residues involved in stabilizing ligand–protein complexes.

Amino Acid Residue	Distance, Å^3^	Bond Category	Bond Type
**Ritanserin in cavity 127 Å^3^**			
**GLU166**	5.29773	Electrostatic	Attractive Charge
**ALA146**	3.65885	Hydrogen Bond; Halogen	Conventional Hydrogen Bond; Halogen (Fluorine)
**MET142**	3.19895	Halogen	Halogen (Fluorine)
**ASP152**	3.68042	Halogen	Halogen (Fluorine)
**LEU193**	2.63495	Halogen	Halogen (Fluorine)
**ALA146**	3.9193	Hydrophobic	Pi–Sigma
**MET163**	3.94403	Hydrophobic	Pi–Sigma
**LEU193**	3.93009	Hydrophobic	Pi–Sigma
**MET163**	3.77279	Other	Pi–Sulfur
**TRP151**	4.06681	Hydrophobic	Pi–Pi Stacked
**TRP151**	4.54522	Hydrophobic	Pi–Pi Stacked
**LEU156**	5.35624	Hydrophobic	Alkyl
**LEU156**	5.08272	Hydrophobic	Pi–Alkyl
**LEU156**	5.06585	Hydrophobic	Pi–Alkyl
**VAL188**	4.92692	Hydrophobic	Pi–Alkyl
**VAL188**	4.81405	Hydrophobic	Pi–Alkyl
**Substance 3 in cavity 127 Å^3^**			
**TRP151**	3.05971	Hydrogen Bond	Conventional Hydrogen Bond
**LEU193**	3.8508	Hydrophobic	Pi–Sigma
**TRP151**	3.94194	Hydrophobic	Pi–Pi Stacked
**TRP151**	5.00543	Hydrophobic	Pi–Pi Stacked
**TRP151**	5.31489	Hydrophobic	Pi–Pi Stacked
**TRP151**	4.69541	Hydrophobic	Pi–Alkyl
**LEU156**	5.44551	Hydrophobic	Pi–Alkyl
**LEU156**	5.2451	Hydrophobic	Pi–Alkyl
**ALA146**	4.39575	Hydrophobic	Pi–Alkyl
**LEU156**	5.32117	Hydrophobic	Pi–Alkyl
**ALA146**	4.32019	Hydrophobic	Pi–Alkyl
**R59949 in cavity 179 Å^3^**			
**GLU162**	4.64611	Electrostatic	Attractive Charge
**ASP136**	3.18667	Halogen	Halogen (Fluorine)
**ASP168**	3.5015	Halogen	Halogen (Fluorine)
**GLY173**	3.68482	Halogen	Halogen (Fluorine)
**SER174**	3.40189	Halogen	Halogen (Fluorine)
**LYS165**	4.83406	Hydrophobic	Alkyl
**VAL135**	4.64342	Hydrophobic	Pi–Alkyl
**Substance 18 in cavity 179 Å^3^**			
**SER132**	2.12242	Hydrogen Bond	Conventional Hydrogen Bond
**ASP136**	3.67142	Electrostatic	Pi–Anion
**ASP136**	3.69271	Electrostatic	Pi–Anion
**LYS165**	4.12278	Hydrophobic	Alkyl
**VAL135**	4.17437	Hydrophobic	Pi–Alkyl
**VAL135**	4.98862	Hydrophobic	Pi–Alkyl
**R59949 in cavity 97 Å^3^**			
**GLU166**	4.80272	Electrostatic	Attractive Charge
**ARG182**	3.12515	Hydrogen Bond; Halogen	Conventional Hydrogen Bond; Halogen (Fluorine)
**GLU166**	3.7357	Hydrogen Bond	Conventional Hydrogen Bond
**GLU166**	3.22464	Hydrogen Bond	Carbon Hydrogen Bond
**ILE167**	3.31873	Halogen	Halogen (Fluorine)
**GLU179**	3.25959	Halogen	Halogen (Fluorine)
**MET163**	3.82172	Other	Pi–Sulfur
**MET163**	3.91728	Other	Pi–Sulfur
**LEU193**	5.31213	Hydrophobic	Pi–Alkyl
**ARG182**	5.20158	Hydrophobic	Pi–Alkyl
**ALA183**	4.29659	Hydrophobic	Pi–Alkyl
**Substance 40 in cavity 97 Å^3^**			
**GLU166**	1.85385	Hydrogen Bond	Conventional Hydrogen Bond
**GLU166**	3.92296	Electrostatic	Pi–Anion
**THR187**	3.27552	Hydrogen Bond	Pi–Donor Hydrogen Bond
**MET163**	3.86247	Hydrophobic	Pi–Sigma
**ALA183**	3.7804	Hydrophobic	Pi–Sigma
**MET163**	4.04032	Other	Pi–Sulfur
**ARG182, ALA183**	4.76159	Hydrophobic	Amide–Pi Stacked
**ILE167**	5.38614	Hydrophobic	Pi–Alkyl
**ARG182**	4.85254	Hydrophobic	Pi–Alkyl
**VAL188**	4.54636	Hydrophobic	Pi–Alkyl
**VAL188**	5.13921	Hydrophobic	Pi–Alkyl
**R59949 in cavity 121 Å^3^**			
**TYR122**	4.03824	Hydrogen Bond	Pi–Donor Hydrogen Bond
**TYR122**	3.51351	Hydrogen Bond	Pi–Donor Hydrogen Bond
**TYR122**	4.19688	Hydrophobic	Pi–Pi Stacked
**TYR148**	3.86497	Hydrophobic	Pi–Pi Stacked
**LEU121**	5.26796	Hydrophobic	Pi–Alkyl
**VAL145**	4.71276	Hydrophobic	Pi–Alkyl
**Substance 28 in cavity 121 Å^3^**			
**GLN141**	3.15959	Hydrogen Bond	Conventional Hydrogen Bond
**VAL145**	3.68904	Hydrogen Bond	Carbon Hydrogen Bond
**ARG144**	4.65278	Electrostatic	Pi–Cation
**GLN141**	3.87404	Hydrogen Bond	Pi–Donor Hydrogen Bond
**VAL145**	3.69747	Hydrophobic	Pi–Sigma
**VAL145**	5.03024	Hydrophobic	Alkyl
**LEU149**	5.09337	Hydrophobic	Alkyl
**LEU149**	5.04472	Hydrophobic	Alkyl
**ARG144**	3.97846	Hydrophobic	Pi–Alkyl
**ARG144**	4.07433	Hydrophobic	Pi–Alkyl
**R59022 in cavity 146 Å^3^**			
**THR124**	2.99368	Hydrogen Bond; Halogen	Conventional Hydrogen Bond; Halogen (Fluorine)
**LEU121**	3.16241	Hydrogen Bond	Carbon Hydrogen Bond
**intermolecular**	3.25258	Hydrogen Bond	Carbon Hydrogen Bond
**TYR122**	3.48925	Hydrogen Bond	Carbon Hydrogen Bond
**LYS137**	4.93829	Electrostatic	Pi–Cation
**THR124**	4.06017	Hydrogen Bond	Pi–Donor Hydrogen Bond
**LEU121**	3.60325	Hydrophobic	Pi–Sigma
**LEU121**	3.94699	Hydrophobic	Pi–Sigma
**THR124**	3.40756	Hydrophobic	Pi–Sigma
**LYS137**	3.22303	Hydrophobic	Pi–Sigma
**LYS137**	4.00354	Hydrophobic	Pi–Alkyl
**Substance 33 in cavity 146 Å^3^**			
**ARG126**	3.19588	Hydrogen Bond	Conventional Hydrogen Bond
**LYS120**	3.8771	Hydrophobic	Alkyl
**ARG126**	4.81701	Hydrophobic	Alkyl
**ARG126**	4.62315	Hydrophobic	Alkyl
**LYS120**	4.5638	Hydrophobic	Pi–Alkyl
**ARG126**	4.95772	Hydrophobic	Pi–Alkyl

Note: The background is used for better perception of information. Colors used according to the bond representation in Figure 4 and Figure 5. **Ritanserin** (6-(2-(4-(*bis*(4-fluorophenyl)methylene)piperidin-1-yl)ethyl)-7-methyl-5*H*-thiazolo[3,2-*a*]pyrimidin-5-one); **3** (2′-(furan-2-yl)-6′*H*-spiro[cyclobutane-1,5′-[1,2,4]triazolo[1,5-*c*]quinazoline]); **R59949** (3-(2-(4-(*bis*(4-fluorophenyl)methylene)piperidin-1-yl)ethyl)-2-thioxo-2,3-dihydroquinazo-lin-4(1*H*)-one); **18** (2′-(1*H*-indol-2-yl)-6′*H*-spiro[cyclopentane-1,5′-[1,2,4]triazolo[1,5-*c*]quinazoline]); **40** (2′-(1*H*-indol-2-yl)-1-methyl-6′*H*-spiro[piperidine-4,5′-[1,2,4]triazolo[1,5-*c*]quinazoline]); **28** (2′-(benzofuran-2-yl)-4-(*tert*-butyl)-6′*H*-spiro[cyclohexane-1,5′-[1,2,4]triazolo[1,5-*c*]quinazoline]); **R59022** (6-(2-(4-((4-fluorophenyl)(phenyl)methylene)piperidin-1-yl)ethyl)-7-methyl-5*H*-thiazolo-[3,2-*a*]pyrimidin-5-one); **33** (2′-((3R,5R)-adamantan-1-yl)-1-methyl-6′*H*-spiro[piperidine-4,5′-[1,2,4]-triazolo[1,5-*c*]quinazoline]). Bond distances are reported in Angstroms (Å) and represent the shortest atomic distance between the interacting groups. Smaller distances generally indicate stronger interactions, particularly for hydrogen bonds, where distances below 3.0 Å typically represent strong binding. Electrostatic interactions include attractive charges (between oppositely charged groups) and π–anion/π–cation interactions (between aromatic systems and charged residues). These interactions can contribute significantly to the binding affinity, especially in polar binding pockets. Hydrogen bonds are classified as conventional (between typical hydrogen bond donors and acceptors), carbon hydrogen bonds (weaker interactions involving C-H as donors), and π–donor hydrogen bonds (where aromatic systems serve as acceptors). Conventional hydrogen bonds with distances under 3.0 Å provide substantial binding energy. Halogen interactions involve fluorine atoms functioning as weak hydrogen bond acceptors or participating in halogen bonding. Though individually weaker than hydrogen bonds, multiple halogen interactions can collectively contribute significant binding energy. Hydrophobic interactions include alkyl (aliphatic–aliphatic), π–alkyl (aromatic–aliphatic), π–sigma, and π–π stacked (aromatic–aromatic) contacts. These non-polar interactions are particularly important in binding pockets with hydrophobic characteristics. Pi–sulfur interactions involve the interaction between aromatic π-systems and sulfur-containing residues (typically methionine), representing a specialized type of non-covalent interaction that can significantly enhance binding stability.

**Table 4:** Drug-likeness assessment according to common medicinal chemistry filters.

Sub	Lipinski	Ghose	Veber	Egan	Muegge
**1–10, 12–19, 21, 23, 24, 31–40**	Yes; 0 violations	Yes	Yes	Yes	Yes
**11, 25, 29**	Yes; 1 violation: MLOGP > 4.15				No; 1 violation: XLOGP3 > 5
**20, 22, 27, 30**	Yes; 0 violations				No; 1 violation: XLOGP3 > 5
**26**	Yes; 1 violation: MLOGP > 4.15	No; 1 violation: WLOGP > 5.6			No; 1 violation: XLOGP3 > 5
**28**	Yes; 1 violation: MLOGP > 4.15	No; 1 violation: WLOGP > 5.6		No; 1 violation: WLOGP > 5.88	No; 1 violation: XLOGP3 > 5
**BMS502**	Yes; 1 violation: MW > 500	No; 2 violations: MW > 480, MR > 130	Yes		
**R59022**	Yes; 1 violation: MLOGP > 4.15	No; 1 violation: MR > 130	Yes		No; 1 violation: XLOGP3 > 5
**R59949**	Yes; 1 violation: MLOGP > 4.1	No; 3 violations: MW > 480, WLOGP > 5.6, MR > 130	Yes	No; 1 violation: WLOGP > 5.88	No; 1 violation: XLOGP3 > 5
**Ritanserin**	Yes; 1 violation: MLOGP > 4.15	No; 2 violations: WLOGP > 5.6, MR > 130	Yes	No; 1 violation: WLOGP > 5.88	No; 1 violation: XLOGP3 > 5

Note: The background is used for better perception of information. “Yes” indicates that the compound passes all criteria for the respective filter, while “No” indicates a violation. The specific violated parameter is indicated after each violation. MLOGP, WLOGP, and XLOGP3 represent different computational methods for calculating the compound’s octanol–water partition coefficient (logP), a measure of lipophilicity. MW = molecular weight; MR = molar refractivity. Compounds with 0–1 violations of Lipinski’s Rule of Five are considered to have favorable drug-like properties.

**Table 5:** Binding affinity comparison between selected patent compounds and spiro derivatives in DGK-α (RCSB PDB ID: 6IIE).

Cavity	Main Pat. Scaffold	p453	13	18	33	40
**Vina scores, kcal/mol**						
**179 Å^3^**	−6.2	−7.1	−8.2	−8.5	−8.2	−7.9
**146 Å^3^**	−5.7	−7.5	−8.4	−7.9	−8.3	−7.8
**127 Å^3^**	−5.3	−7.0	−7.5	−7.9	−7.2	−8.2
**121 Å^3^**	−4.8	−6.6	−6.8	−6.9	−7.5	−6.8
**97 Å^3^**	−4.6	−6.6	−6.9	−6.6	−7.2	−7.1

Note: The background is used for better perception of information. Binding affinity values represent the Vina scores (kcal/mol) obtained from molecular docking studies using CB-Dock2, with more negative values indicating a stronger predicted binding affinity. **Main pat. scaffold** refers to *N*-methyl[1,2,4]triazolo[4,3-*a*]quinazolin-5-amine, representing the basic scaffold from patent CN 115362003 B [10]. Compound **p453** refers to (8-chloro-5-(5-(cyclopropylethynyl)-3,4-dihydroquinolin-1(2*H*)-yl)-7-fluoro-[1,2,4]triazolo[4,3-*a*]quinazoline), which allows for a correlation with the compound with the highest reported activity.

## Data Availability

The data presented in this study are available upon request from the corresponding author. The Supplementary Materials are available free of charge on the MDPI Publication website.

## Supplementary Materials

The following supporting information can be downloaded at https://www.mdpi.com/article/10.3390/molecules30112324/s1: Table S1. Physicochemical properties and molecular descriptors. Table S2. In silico oral toxicity predictions for spiro[1,2,4]triazolo[1,5-*c*]quinazoline derivatives using Protox-2 and 3. Table S3. Lipophilicity parameters using different methods. Table S4. Solubility analysis in different solvent systems. Table S5. Pharmacokinetic and enzyme inhibitory properties of the compounds. Table S6. Bioavailability assessment and drug-likeness parameters.

## Abbreviations

The following abbreviations are used in this manuscript:

DGK-α	Diacylglycerol kinase alpha
PDB	Protein Data Bank
EF	EF-hand (calcium-binding motif)
DAG	Diacylglycerol
PA	Phosphatidic acid
PKC	Protein kinase C
PH	Pleckstrin homology (domain)
SAM	Sterile alpha motif
MARCKS	Myristoylated alanine-rich C-kinase substrate
PDZ	Postsynaptic density protein, Drosophila disc large tumor suppressor, Zonula occludens-1 protein (domain)
HIV	Human immunodeficiency virus
HBV	Hepatitis B virus
5-HT2R	Serotonin 2 receptor
IC_50_	Half maximal inhibitory concentration
Kd	Dissociation constant
ADME	Absorption, eistribution, metabolism, excretion
TPSA	Topological polar surface area
ESOL	Estimated aqueous solubility
MW	Molecular weight
MR	Molecular refractivity
logP	Partition coefficient (octanol–water)
PAINS	Pan-Assay Interference Compounds
BBB	Blood–brain barrier
P-gp	P-glycoprotein
CYP	Cytochrome P450
LD50	Median lethal dose
HT	Hepatotoxicity
CG	Carcinogenicity
IT	Immunotoxicity
MG	Mutagenicity
CT	Cytotoxicity
SAR	Structure–activity relationship
CNS	Central nervous system
PMA	Phorbol 12-myristate 13-acetate
GHS	Globally Harmonized System (of Classification and Labeling of Chemicals)
