# Supplementary material for: In Silico Identification and Characterization of Spiro[1,2,4]triazolo[1,5-c]quinazolines as Diacylglycerol Kinase α Modulators
Source: Molecules. 2025 May 26;30(11):2324. doi: 10.3390/molecules30112324 (PMC12156019; doi:10.3390/molecules30112324)
Supplement: Supplementary file 1 [file molecules-30-02324-s001.zip › molecules-3630966-supplementary.pdf]

SUPPLEMENTARY MATERIAL

In Silico Identification and Characterization of Spiro[1,2,4]triazolo[1,5-c]quinazolines as Diacylglycerol Kinase α Modulators

Lyudmyla Antypenko [1,*], Kostiantyn Shabelnyk [2], Oleksii Antypenko [2], Mieko Arisawa [3], Oleksandr Kamyshnyi [4,*], Valentyn Oksenych [5,*], and Serhii Kovalenko [6]

1 Independent Researcher, 11 Lamana Str., 69063 Zaporizhzhia, Ukraine
2 Department of Pharmaceutical, Organic and Bioorganic Chemistry, Zaporizhzhia State Medical and Pharmaceutical University, 26 Maria Prymachenko Blvd., 69035 Zaporizhzhia, Ukraine; kshabelnik@gmail.com (K.S.); antypenkoan@gmail.com (O.A.)
3 Graduate School of Bioresources and Bioenvironmental Sciences, Kyushu University, 744 W5-674, Motooka Nishi-ku, Fukuoka 819-0395, Japan; arisawa@agr.kyushu-u.ac.jp
4 Department of Microbiology, Virology and Immunology, I. Horbachevsky Ternopil State Medical University, 46001 Ternopil, Ukraine
5 Department of Clinical Science, University of Bergen, Langes Gate 1-3, 5020 Bergen, Norway
6 Institute of Chemistry and Geology, Oles Honchar Dnipro National University, 72 Nauky Ave., 49010 Dnipro, Ukraine; kovalenkosergiy@gmail.com
* Correspondence: antypenkol@gmail.com (L.A.); kamyshnyi_om@tdmu.edu.ua (O.K.); valentyn.oksenych@uib.no (V.O.)

Table S1. Physicochemical properties and molecular descriptors.

Sub.	MW, g/mol	HA	AHA	Csp^3	RB	NBA	NBD	Mol. refr.	TPSA, Å²
**1**	252.31	19	11	0.43	1	2	1	76.70	42.74
**2**	294.39	22	11	0.56	1	2	1	91.12	42.74
**3**	278.31	21	16	0.25	1	3	1	81.93	55.88
**4**	328.37	25	20	0.20	1	3	1	99.44	55.88
**5**	294.37	21	16	0.25	1	2	1	87.54	70.98
**6**	289.33	22	17	0.24	1	3	1	87.46	55.63
**7**	289.33	22	17	0.24	1	3	1	87.46	55.63
**8**	327.38	25	20	0.20	1	2	2	101.52	58.53
**9**	266.34	20	11	0.50	1	2	1	81.50	42.74
**10**	308.42	23	11	0.58	1	2	1	95.92	42.74
**11**	360.50	27	11	0.65	1	2	1	110.81	42.74
**12**	292.34	22	16	0.29	1	3	1	86.74	55.88
**13**	342.39	26	20	0.24	1	3	1	104.24	55.88
**14**	308.40	22	16	0.29	1	2	1	92.35	70.98
**15**	303.36	23	17	0.28	1	3	1	92.27	55.63
**16**	303.36	23	17	0.28	1	3	1	92.27	55.63
**17**	303.36	23	17	0.28	1	3	1	92.27	55.63
**18**	341.41	26	20	0.24	1	2	2	106.33	58.53
**19**	280.37	21	11	0.53	1	2	1	86.31	42.74
**20**	322.45	24	11	0.60	1	2	1	100.73	42.74
**21**	306.36	23	16	0.33	1	3	1	91.55	55.88
**22**	356.42	27	20	0.27	1	3	1	109.05	55.88
**23**	322.43	23	16	0.33	1	2	1	97.16	70.98
**24**	317.39	24	17	0.32	1	3	1	97.07	55.63
**25**	336.47	25	11	0.62	2	2	1	105.28	42.74
**26**	378.55	28	11	0.67	2	2	1	119.70	42.74
**27**	362.47	27	16	0.45	2	3	1	110.51	55.88
**28**	412.53	31	20	0.38	2	3	1	128.02	55.88
**29**	378.53	27	16	0.45	2	2	1	116.12	70.98
**30**	373.49	28	17	0.43	2	3	1	116.04	55.63
**31**	295.38	22	11	0.53	1	3	1	93.12	45.98
**32**	337.46	25	11	0.60	1	3	1	107.54	45.98
**33**	389.54	29	11	0.67	1	3	1	122.42	45.98
**34**	321.38	24	16	0.33	1	4	1	98.36	59.12
**35**	371.44	28	20	0.27	1	4	1	115.86	59.12
**36**	337.44	24	16	0.33	1	3	1	103.97	74.22
**37**	332.40	25	17	0.32	1	4	1	103.89	58.87
**38**	332.40	25	17	0.32	1	4	1	103.89	58.87
**39**	332.40	25	17	0.32	1	4	1	103.89	58.87
**40**	370.45	28	20	0.27	1	3	2	117.95	61.77
**BMS_502**	516.50	38	22	0.22	5	8	0	145.95	110.98
**R59022**	459.58	33	21	0.26	5	4	0	136.87	65.85
**R59949**	489.58	35	22	0.21	5	4	1	142.21	73.12
**Ritanserin**	477.57	34	21	0.26	5	5	0	136.82	65.85

MW - Molecular weight; HA - Heavy atoms; AHA - Aromatic heavy atoms; Csp^3 - Fraction of sp^3 hybridized carbon atoms; RB - Rotatable bonds; NBA - Number of hydrogen bond acceptors; NBD - Number of hydrogen bond donors; Mol. refr. - Molecular refractivity; TPSA - Topological polar surface area.

Table S2. *In silico* oral toxicity predictions for spiro[1,2,4]triazolo[1,5-*c*]quinazoline derivatives using Protox- 2 and 3.

Sub.*	Oral toxicity			Prediction: active, probability from (Yes/No) **				
	Toxicity index*	LD_{50}, mg/kg	Prediction accuracy, %	HT	CG	IT	MG	CT
**1**	IV	2000	54.26	0.56/no	0.55/yes	0.96/no	0.50/no	0.60/yes
**2**		2000	54.26	0.64/no	0.54/yes	0.97/no	0.55/no	0.60/yes
**3**		2000	54.26	0.52/no	0.56/yes	0.97/no	0.54/no	0.59/yes
**4**		2000	54.26	0.52/no	0.56/yes	0.98/no	0.54/no	0.59/yes
**5**		900	23.00	0.55/no	0.51/no	0.99/no	0.56/no	0.56/yes
**6**		1200	23.00	0.59/no	0.55/yes	0.89/no	0.55/no	0.60/yes
**7**		1000	54.26	0.59/no	0.55/yes	0.97/no	0.55/no	0.60/yes
**8**		1200	23.00	0.57/no	0.54/yes	0.80/no	0.56/no	0.60/yes
**9**		1200	54.26	0.59/no	0.55/yes	0.97/no	0.50/no	0.60/yes
**10**		2000	54.26	0.65/no	0.53/yes	0.98/no	0.55/no	0.59/yes
**11**		2000	54.26	0.65/no	0.53/yes	0.99/no	0.57/no	0.59/yes
**12**		2000	54.26	0.55/no	0.55/yes	0.98/no	0.54/no	0.59/yes
**13**		2000	54.26	0.55/no	0.55/yes	0.99/no	0.54/no	0.59/yes
**14**		900	23.00	0.58/no	0.51/no	0.99/no	0.56/no	0.55/yes
**15**		1200	23.00	0.62/no	0.55/yes	0.93/no	0.55/no	0.59/yes
**16**		1000	54.26	0.62/no	0.55/yes	0.98/no	0.55/no	0.59/yes
**17**	V	2400	23.00	0.62/no	0.55/yes	0.99/no	0.55/no	0.59/yes
**18**	IV	1200	23.00	0.60/no	0.54/yes	0.88/no	0.56/no	0.59/yes
**19**		2000	54.26	0.62/no	0.53/yes	0.97/no	0.50/no	0.59/yes
**20**		2000	54.26	0.65/no	0.53/yes	0.98/no	0.55/no	0.59/yes
**21**		2000	54.26	0.58/no	0.53/yes	0.98/no	0.56/no	0.58/yes
**22**		2000	54.26	0.58/no	0.53/yes	0.99/no	0.56/no	0.58/yes
**23**		1200	23.00	0.61/no	0.53/yes	0.99/no	0.56/no	0.55/yes
**24**		1000	54.26	0.65/no	0.53/yes	0.98/no	0.55/no	0.59/yes
**25**		2000	54.26	0.63/no	0.50/no	0.92/no	0.59/no	0.53/no
**26**		2000	54.26	0.65/no	0.50/no	0.95/no	0.61/no	0.53/no
**27**		2000	54.26	0.58/no	0.50/yes	0.93/no	0.61/no	0.53/no
**28**		2000	54.26	0.58/no	0.50/yes	0.96/no	0.61/no	0.53/no
**29**		1200	23.00	0.61/no	0.56/no	0.99/no	0.59/no	0.57/no
**30**		1000	54.26	0.65/no	0.50/no	0.92/no	0.61/no	0.53/no
**31**		1200	54.26	0.73/no	0.50/no	0.96/no	0.57/no	0.60/yes
**32**		1200	54.26	0.76/no	0.53/no	0.97/no	0.61/no	0.60/yes
**33**		1200	54.26	0.76/no	0.53/no	0.99/no	0.61/no	0.60/yes
**34**		2000	54.26	0.66/no	0.50/no	0.97/no	0.59/no	0.60/yes
**35**		1460	54.26	0.66/no	0.50/no	0.98/no	0.59/no	0.60/yes
**36**		900	23.00	0.69/no	0.57/no	0.99/no	0.60/no	0.56/yes
**37**		440	23.00	0.76/no	0.53/no	0.90/no	0.61/no	0.60/yes
**38**		1000	23.00	0.76/no	0.53/no	0.97/no	0.61/no	0.60/yes
**39**		1000	23.00	0.76/no	0.53/no	0.99/no	0.61/no	0.60/yes
**40**		440	23.00	0.72/no	0.53/no	0.82/no	0.62/no	0.60/yes
**BMS502**		1600	54.26	0.68/no	0.50/no	0.84/no	0.91/yes	0.58/no
**R59022**		1000	54.26	0.68/no	0.63/no	0.99/no	0.62/no	0.67/no
**R59949**		900	67.38	0.57/no	0.63/no	0.75/no	0.62/no	0.74/no
**Ritanserin**		1000	54.26	0.68/no	0.63/no	0.99/no	0.62/no	0.67/no

* Class IV: harmful if swallowed ($300 < LD_{50} \leq 2000$); Class V: may be harmful if swallowed ($2000 < LD_{50} \leq 5000$).

** – HT – Hepatotoxicity, CG - Carcinogenicity, IT - Immunotoxicity, MG - Mutagenicity, CT - Cytotoxicity

Table S3. Lipophilicity parameters using different methods.

Sub.	iLOGP	XLOGP3	WLOGP	MLOGP	SILICOS-IT	Consensus
**37**	2.90	2.47	1.75	2.10	1.69	2.18
**38**	2.95	2.43	1.75	2.10	1.69	2.18
**39**	2.93	2.43	1.75	2.10	1.69	2.18
**31**	2.98	2.41	1.50	2.41	1.68	2.20
**34**	3.16	2.60	1.95	1.90	1.65	2.25
**6**	2.71	2.78	2.59	2.44	2.28	2.56
**1**	2.77	2.73	2.34	2.75	2.28	2.57
**7**	2.81	2.75	2.59	2.44	2.28	2.57
**3**	2.89	2.92	2.79	2.24	2.25	2.62
**15**	2.82	3.14	2.98	2.68	2.50	2.82
**16**	2.89	3.11	2.98	2.68	2.50	2.83
**17**	2.86	3.11	2.98	2.68	2.50	2.83
**9**	2.90	3.09	2.73	3.00	2.50	2.84
**12**	2.97	3.28	3.18	2.48	2.46	2.87
**36**	3.25	3.22	2.42	2.72	2.90	2.90
**40**	3.22	3.66	2.84	2.89	2.75	3.07
**24**	3.06	3.65	3.37	2.91	2.72	3.14
**19**	3.11	3.63	3.12	3.24	2.72	3.16
**32**	3.58	4.04	2.74	3.11	2.35	3.16
**21**	3.20	3.82	3.57	2.72	2.68	3.20
**35**	3.63	3.94	3.10	2.89	2.62	3.24
**5**	3.06	3.54	3.26	3.07	3.49	3.28
**8**	3.00	3.98	3.68	3.26	3.34	3.45
**2**	3.32	4.36	3.58	3.48	2.94	3.53
**14**	3.16	3.89	3.65	3.31	3.71	3.54
**4**	3.38	4.26	3.94	3.26	3.21	3.61
**18**	3.09	4.34	4.07	3.48	3.56	3.71
**33**	3.87	4.72	3.16	3.98	2.83	3.71
**10**	3.47	4.72	3.97	3.71	3.17	3.81
**13**	3.41	4.61	4.33	3.48	3.43	3.85
**23**	3.37	4.43	4.04	3.55	3.93	3.86
**20**	3.62	5.26	4.36	3.94	3.39	4.11
**30**	3.78	5.09	4.64	3.80	3.61	4.18
**22**	3.79	5.16	4.72	3.70	3.65	4.21
**25**	3.82	5.07	4.39	4.16	3.58	4.21
**27**	3.93	5.26	4.84	3.62	3.56	4.24
**11**	3.79	5.40	4.39	4.59	3.64	4.36
**29**	4.10	5.88	5.31	4.45	4.81	4.91
**26**	4.31	6.70	5.63	4.80	4.29	5.14
**28**	4.35	6.60	5.99	4.54	4.57	5.21
**BMS_502**	3.52	3.62	3.66	2.49	2.34	3.13
**R59022**	4.45	5.10	5.38	4.82	6.66	5.28
**R59949**	4.46	6.30	6.39	4.87	7.57	5.92
**Ritanserin**	4.59	5.20	5.94	5.18	7.08	5.60

Values represent predicted partition coefficients (logP) calculated using five different computational methods: iLOGP (internal VCCLAB method), XLOGP3 (atom-based method), WLOGP (atomistic method based on fragmental data), MLOGP (topological method), and SILICOS-IT (hybrid method).
Consensus values represent the arithmetic mean of the five calculated logP values for each compound.
Compounds are ordered by increasing consensus logP values.
Higher logP values indicate greater lipophilicity, suggesting enhanced blood-brain barrier penetration potential for CNS-targeted compounds.

Table S4. Solubility analysis in different solvent systems.

Sub.	ESOL	mg/ml; mol/l	Class	Ali	mg/ml; mol/l	Class	SILI-COS-IT	mg/ml; mol/l	Class
**1**	-3.49	8.23e-02 mg/ml ; 3.26e-04 mol/l	S	-3.28	1.32e-01 mg/ml ; 5.23e-04 mol/l	S	-4.3	1.26e-02 mg/ml ; 5.00e-05 mol/l	MS
**31**	-3.49	9.48e-02 mg/ml ; 3.21e-04 mol/l	S	-3.02	2.84e-01 mg/ml ; 9.60e-04 mol/l	S	-4.29	1.51e-02 mg/ml ; 5.13e-05 mol/l	MS
**9**	-3.78	4.43e-02 mg/ml ; 1.66e-04 mol/l	S	-3.66	5.89e-02 mg/ml ; 2.21e-04 mol/l	S	-4.58	7.05e-03 mg/ml ; 2.65e-05 mol/l	MS
**7**	-3.87	3.88e-02 mg/ml ; 1.34e-04 mol/l	S	-3.57	7.73e-02 mg/ml ; 2.67e-04 mol/l	S	-5.83	4.23e-04 mg/ml ; 1.46e-06 mol/l	MS
**38**	-3.87	4.49e-02 mg/ml ; 1.35e-04 mol/l	S	-3.31	1.63e-01 mg/ml ; 4.91e-04 mol/l	S	-5.82	5.08e-04 mg/ml ; 1.53e-06 mol/l	MS
**39**	-3.87	4.49e-02 mg/ml ; 1.35e-04 mol/l	S	-3.31	1.63e-01 mg/ml ; 4.91e-04 mol/l	S	-5.82	5.08e-04 mg/ml ; 1.53e-06 mol/l	MS
**6**	-3.89	3.72e-02 mg/ml ; 1.29e-04 mol/l	S	-3.6	7.20e-02 mg/ml ; 2.49e-04 mol/l	S	-5.83	4.23e-04 mg/ml ; 1.46e-06 mol/l	MS
**37**	-3.89	4.24e-02 mg/ml ; 1.28e-04 mol/l	S	-3.35	1.48e-01 mg/ml ; 4.46e-04 mol/l	S	-5.82	5.08e-04 mg/ml ; 1.53e-06 mol/l	MS
**3**	-3.9	3.48e-02 mg/ml ; 1.25e-04 mol/l	S	-3.75	4.90e-02 mg/ml ; 1.76e-04 mol/l	S	-5.42	1.05e-03 mg/ml ; 3.76e-06 mol/l	MS
**34**	-3.9	4.07e-02 mg/ml ; 1.27e-04 mol/l	S	-3.49	1.04e-01 mg/ml ; 3.23e-04 mol/l	S	-5.41	1.25e-03 mg/ml ; 3.90e-06 mol/l	MS
**16**	-4.16	2.09e-02 mg/ml ; 6.90e-05 mol/l	MS	-3.95	3.43e-02 mg/ml ; 1.13e-04 mol/l	S	-6.11	2.37e-04 mg/ml ; 7.80e-07 mol/l	PS
**17**	-4.16	2.09e-02 mg/ml ; 6.90e-05 mol/l	MS	-3.95	3.43e-02 mg/ml ; 1.13e-04 mol/l	S	-6.11	2.37e-04 mg/ml ; 7.80e-07 mol/l	PS
**15**	-4.18	2.00e-02 mg/ml ; 6.61e-05 mol/l	MS	-3.98	3.19e-02 mg/ml ; 1.05e-04 mol/l	S	-6.11	2.37e-04 mg/ml ; 7.80e-07 mol/l	PS
**12**	-4.19	1.88e-02 mg/ml ; 6.44e-05 mol/l	MS	-4.13	2.18e-02 mg/ml ; 7.44e-05 mol/l	MS	-5.7	5.85e-04 mg/ml ; 2.00e-06 mol/l	MS
**19**	-4.19	1.82e-02 mg/ml ; 6.50e-05 mol/l	MS	-4.22	1.71e-02 mg/ml ; 6.09e-05 mol/l	MS	-4.85	3.95e-03 mg/ml ; 1.41e-05 mol/l	MS
**5**	-4.39	1.19e-02 mg/ml ; 4.04e-05 mol/l	MS	-4.72	5.67e-03 mg/ml ; 1.93e-05 mol/l	MS	-5.47	9.86e-04 mg/ml ; 3.35e-06 mol/l	MS
**36**	-4.39	1.38e-02 mg/ml ; 4.09e-05 mol/l	MS	-4.45	1.19e-02 mg/ml ; 3.54e-05 mol/l	MS	-5.45	1.19e-03 mg/ml ; 3.52e-06 mol/l	MS

**24**	-4.57	8.63e-03 mg/ml ; 2.72e-05 mol/l	MS	-4.51	9.88e-03 mg/ml ; 3.11e-05 mol/l	MS	-6.38	1.32e-04 mg/ml ; 4.17e-07 mol/l	PS
**21**	-4.59	7.79e-03 mg/ml ; 2.54e-05 mol/l	MS	-4.69	6.27e-03 mg/ml ; 2.05e-05 mol/l	MS	-5.97	3.27e-04 mg/ml ; 1.07e-06 mol/l	MS
**14**	-4.67	6.52e-03 mg/ml ; 2.11e-05 mol/l	MS	-5.08	2.57e-03 mg/ml ; 8.35e-06 mol/l	MS	-5.75	5.52e-04 mg/ml ; 1.79e-06 mol/l	MS
**2**	-4.72	5.66e-03 mg/ml ; 1.92e-05 mol/l	MS	-4.97	3.13e-03 mg/ml ; 1.06e-05 mol/l	MS	-5.12	2.21e-03 mg/ml ; 7.50e-06 mol/l	MS
**32**	-4.74	6.18e-03 mg/ml ; 1.83e-05 mol/l	MS	-4.71	6.59e-03 mg/ml ; 1.95e-05 mol/l	MS	-5.11	2.65e-03 mg/ml ; 7.85e-06 mol/l	MS
**8**	-4.9	4.09e-03 mg/ml ; 1.25e-05 mol/l	MS	-4.91	4.02e-03 mg/ml ; 1.23e-05 mol/l	MS	-7.08	2.75e-05 mg/ml ; 8.41e-08 mol/l	PS
**40**	-4.91	4.61e-03 mg/ml ; 1.24e-05 mol/l	MS	-4.65	8.36e-03 mg/ml ; 2.26e-05 mol/l	MS	-7.05	3.32e-05 mg/ml ; 8.95e-08 mol/l	PS
**10**	-5.01	2.99e-03 mg/ml ; 9.69e-06 mol/l	MS	-5.35	1.39e-03 mg/ml ; 4.50e-06 mol/l	MS	-5.4	1.24e-03 mg/ml ; 4.01e-06 mol/l	MS
**23**	-5.08	2.69e-03 mg/ml ; 8.34e-06 mol/l	MS	-5.64	7.41e-04 mg/ml ; 2.30e-06 mol/l	MS	-6.02	3.09e-04 mg/ml ; 9.57e-07 mol/l	PS
**4**	-5.09	2.70e-03 mg/ml ; 8.21e-06 mol/l	MS	-5.15	2.35e-03 mg/ml ; 7.16e-06 mol/l	MS	-7.08	2.75e-05 mg/ml ; 8.38e-08 mol/l	PS
**35**	-5.09	3.04e-03 mg/ml ; 8.17e-06 mol/l	MS	-4.88	4.88e-03 mg/ml ; 1.31e-05 mol/l	MS	-7.05	3.30e-05 mg/ml ; 8.89e-08 mol/l	PS
**18**	-5.19	2.18e-03 mg/ml ; 6.39e-06 mol/l	MS	-5.28	1.78e-03 mg/ml ; 5.20e-06 mol/l	MS	-7.34	1.54e-05 mg/ml ; 4.52e-08 mol/l	PS
**25**	-5.31	1.63e-03 mg/ml ; 4.85e-06 mol/l	MS	-5.71	6.56e-04 mg/ml ; 1.95e-06 mol/l	MS	-5.8	5.30e-04 mg/ml ; 1.57e-06 mol/l	MS
**13**	-5.37	1.46e-03 mg/ml ; 4.26e-06 mol/l	MS	-5.51	1.06e-03 mg/ml ; 3.10e-06 mol/l	MS	-7.35	1.54e-05 mg/ml ; 4.49e-08 mol/l	PS
**20**	-5.43	1.21e-03 mg/ml ; 3.75e-06 mol/l	MS	-5.91	3.99e-04 mg/ml ; 1.24e-06 mol/l	MS	-5.67	6.91e-04 mg/ml ; 2.14e-06 mol/l	MS
**33**	-5.44	1.40e-03 mg/ml ; 3.60e-06 mol/l	MS	-5.41	1.50e-03 mg/ml ; 3.85e-06 mol/l	MS	-5.7	7.84e-04 mg/ml ; 2.01e-06 mol/l	MS
**30**	-5.68	7.81e-04 mg/ml ; 2.09e-06 mol/l	MS	-6	3.72e-04 mg/ml ; 9.97e-07 mol/l	PS	-7.32	1.78e-05 mg/ml ; 4.76e-08 mol/l	PS
**11**	-5.71	6.99e-04 mg/ml ; 1.94e-06 mol/l	MS	-6.05	3.20e-04 mg/ml ; 8.87e-07 mol/l	PS	-6	3.65e-04 mg/ml ; 1.01e-06 mol/l	MS
**27**	-5.71	7.11e-04 mg/ml ; 1.96e-06 mol/l	MS	-6.18	2.38e-04 mg/ml ; 6.56e-07 mol/l	PS	-6.92	4.39e-05 mg/ml ; 1.21e-07 mol/l	PS

**22**	-5.78	5.88e-04 mg/ml ; 1.65e-06 mol/l	MS	-6.08	2.97e-04 mg/ml ; 8.33e-07 mol/l	PS	-7.62	8.61e-06 mg/ml ; 2.42e-08 mol/l	PS
**29**	-6.2	2.40e-04 mg/ml ; 6.34e-07 mol/l	PS	-7.14	2.72e-05 mg/ml ; 7.19e-08 mol/l	PS	-6.96	4.15e-05 mg/ml ; 1.10e-07 mol/l	PS
**26**	-6.57	1.03e-04 mg/ml ; 2.71e-07 mol/l	PS	-7.4	1.50e-05 mg/ml ; 3.97e-08 mol/l	PS	-6.61	9.29e-05 mg/ml ; 2.45e-07 mol/l	PS
**28**	-6.9	5.18e-05 mg/ml ; 1.26e-07 mol/l	PS	-7.57	1.10e-05 mg/ml ; 2.67e-08 mol/l	PS	-8.55	1.16e-06 mg/ml ; 2.81e-09 mol/l	PS
**BMS_502**	-5.42	1.96e-03 mg/ml ; 3.79e-06 mol/l	MS	-5.64	1.19e-03 mg/ml ; 2.30e-06 mol/l	MS	-7.49	1.68e-05 mg/ml ; 3.24e-08 mol/l	PS
**R59022**	-6.04	4.16e-04 mg/ml ; 9.05e-07 mol/l	PS	-6.23	2.73e-04 mg/ml ; 5.94e-07 mol/l	PS	-8.81	7.13e-07 mg/ml ; 1.55e-09 mol/l	PS
**R59949**	-6.98	5.13e-05 mg/ml ; 1.05e-07 mol/l	PS	-7.62	1.16e-05 mg/ml ; 2.38e-08 mol/l	PS	-9.51	1.51e-07 mg/ml ; 3.09e-10 mol/l	PS
**Ritanserin**	-6.20	2.99e-04 mg/ml ; 6.25e-07 mol/l	PS	-6.33	2.23e-04 mg/ml ; 4.68e-07 mol/	PS	-9.07	4.07e-07 mg/ml ; 8.53e-10 mol/l	PS

ESOL, Ali, and SILI-COS-IT represent different solubility prediction models or experimental solvent systems. Values are presented as both mg/ml and mol/l concentrations. Solubility classification codes: HS = Highly Soluble, S = Soluble, MS = Moderately Soluble, PS = Poorly Soluble.

Table S5. Pharmacokinetic and enzyme inhibitory properties compounds.

Sub	GA	BBB	P-gp substr.	Inhibitor CYP1A2	Inhibitor CYP2C19	Inhibitor CYP2C9	Inhibitor CYP2D6	Inhibitor CYP3A4	Log Kp. cm/s
**26**	High	+	+	-	-	+	-	+	-3.85
**28**		-	+	-	+	+	+	-	-4.13
**29**		-	+	+	+	+	+	+	-4.43
**20**		+	+	+	-	+	+	-	-4.53
**11**		+	-	-	+	-	-	+	-4.67
**25**		+	+	-	-	+	+	+	-4.75
**27**		+	+	+	+	+	+	+	-4.78
**22**		+	+	+	+	+	+	-	-4.81
**10**		+	+	+	-	+	+	+	-4.83
**30**		+	+	+	+	+	+	+	-4.96
**2**		+	+	+	-	+	+	-	-5.00
**13**		+	+	+	+	+	+	+	-5.12
**23**		+	+	+	+	+	+	+	-5.12
**4**		+	+	+	+	+	+	+	-5.28
**18**		+	+	+	+	+	+	+	-5.30
**33**		+	-	-	+	-	-	+	-5.32
**14**		+	+	+	+	+	-	+	-5.42
**18**		+	+	+	-	-	+	+	-5.43
**21**		+	+	+	-	+	+	+	-5.46
**8**		+	+	+	-	+	+	+	-5.47
**32**		+	+	+	+	+	+	+	-5.49
**5**		+	+	+	+	+	-	+	-5.58
**24**		+	+	+	-	+	+	+	-5.64
**9**		+	+	+	-	-	+	+	-5.73
**12**		+	+	+	-	+	+	+	-5.75
**35**		+	+	+	-	+	+	+	-5.77
**1**		+	+	+	-	-	+	+	-5.90
**3**		+	+	+	-	+	+	+	-5.92
**15**		+	+	+	-	+	+	+	-5.92
**16**		+	+	+	-	+	+	+	-5.94
**17**		+	+	+	-	+	+	+	-5.94
**40**		+	+	+	-	+	+	+	-5.96
**36**		+	+	+	+	+	+	+	-6.07
**6**		+	+	+	-	+	+	+	-6.09
**7**		+	+	+	-	+	+	+	-6.11
**31**		+	+	+	-	-	+	-	-6.39
**34**		+	+	+	-	+	+	+	-6.41
**37**		+	+	+	-	+	+	+	-6.57
**38**		+	+	+	-	+	+	+	-6.60
**39**		+	+	+	-	+	+	+	-6.60
**BMS_502**		-	+	-	+	+	-	+	-6.88
**R59022**		-	+	-	+	+	-	+	-5.48

**R59949**		-	-	-	+	-	-	-	-4.81
**Ritanserin**		-	+	-	+	+	-	+	-5.52

GIA = Gastro-Intestinal Absorption (High represents high absorption level).
BBB = Blood-Brain Barrier penetration (+ indicates positive binding, - indicates negative binding).
P-gp substr. = P-glycoprotein substrate (+ indicates the compound is a substrate, - indicates it is not).
Inhibitor columns (CYP1A2, CYP2C19, CYP2C9, CYP2D6, CYP3A4) indicate whether the compound inhibits the respective cytochrome P450 enzyme (+ indicates inhibition, - indicates no inhibition).
Log Kp = Logarithm of permeability coefficient in cm/s, indicating the compound's ability to penetrate biological membranes, which correlates with gastro-intestinal absorption.
Compounds are arranged in order of decreasing permeability (increasing negative Log Kp values), which corresponds to decreasing gastro-intestinal absorption efficiency.

Table S6. Bioavailability assessment and drug-likeness parameters.

Sub.	Bioavailability, Abbot	Synthetic assessment of availability	Brenk alert, PAINS	Lead compounds, alerts
**1**	0.55	3.50	No	Yes
**7**		3.50		Yes
**6**		3.51		Yes
**17**		3.52		Yes
**3**		3.53		Yes
**5**		3.58		No; 1 violation: XLOGP3>3.5
**9**		3.59		Yes
**15**		3.59		Yes
**16**		3.59		Yes
**12**		3.61		Yes
**14**		3.67		No; 1 violation: XLOGP3>3.5
**8**		3.68		No; 1 violation: XLOGP3>3.5
**19**		3.69		No; 1 violation: XLOGP3>3.5
**24**		3.69		No; 1 violation: XLOGP3>3.5
**21**		3.72		No; 1 violation: XLOGP3>3.5
**39**		3.74		Yes
**18**		3.77		No; 1 violation: XLOGP3>3.5
**23**		3.77		No; 1 violation: XLOGP3>3.5
**31**		3.79		Yes
**4**		3.80		No; 1 violation: XLOGP3>3.5
**37**		3.80		Yes
**38**		3.80		Yes
**34**		3.82		Yes
**2**		3.85		No; 1 violation: XLOGP3>3.5
**36**		3.87		Yes
**13**		3.88		No; 1 violation: XLOGP3>3.5
**10**		3.94		No; 1 violation: XLOGP3>3.5
**40**		3.98		No; 2 violations: MW>350, XLOGP3>3.5
**22**		3.99		No; 2 violations: MW>350, XLOGP3>3.5
**20**		4.04		No; 1 violation: XLOGP3>3.5
**35**		4.08		No; 2 violations: MW>350, XLOGP3>3.5
**32**		4.15		No; 1 violation: XLOGP3>3.5
**25**		4.85		No; 1 violation: XLOGP3>3.5

**30**		4.86		No; 2 violations: MW>350, XLOGP3>3.5
**27**		4.88		No; 2 violations: MW>350, XLOGP3>3.5
**29**		4.93		No; 2 violations: MW>350, XLOGP3>3.5
**28**		5.16		No; 2 violations: MW>350, XLOGP3>3.5
**26**		5.22		No; 2 violations: MW>350, XLOGP3>3.5
**11**		6.33		No; 2 violations: MW>350, XLOGP3>3.5
**33**		6.55		No; 2 violations: MW>350, XLOGP3>3.5
**BMS_502**		3.81	2 alerts: nitro group , oxygen – nitrogen single bond	No; 2 violations: MW>350, XLOGP3>3.5
**R59022**		3.73	No	No; 2 violations: MW>350, XLOGP3>3.5
**R59949**		3.67	1 alert: thiocarbonyl_group	No; 2 violations: MW>350, XLOGP3>3.5
**Ritanserin**		3.73	No	No; 2 violations: MW>350, XLOGP3>3.5

Bioavailability values are reported according to Abbott's lipophilicity scale where values closer to 1 indicate better oral bioavailability potential.

Synthetic assessment of availability represents a calculated parameter estimating the ease of chemical synthesis, with lower values indicating more favorable synthetic accessibility.

XLOGP3 values represent the calculated octanol-water partition coefficient, with values >3.5 potentially indicating reduced aqueous solubility and increased lipophilicity.

Brenk and PAINS (Pan-Assay Interference Compounds) alerts identify structural features associated with false positives in biochemical screening or problematic pharmacokinetic properties.

Lead compounds assessment includes evaluation using multiple pharmaceutical industry drug-likeness filters: Lipinski (Pfizer), Ghose (Amgen), Veber (GSK), Egan (Pharmacia), and Muegge (Bayer). "Yes" indicates the compound meets all filter criteria with 0 violations. The specific violations noted (e.g., MW<250, XLOGP3>3.5, MW>350) refer to parameters outside the optimal ranges established by these pharmaceutical industry filters.
